# Direct sound printing

**DOI:** 10.1038/s41467-022-29395-1

**Published:** 2022-04-06

**Authors:** Mohsen Habibi, Shervin Foroughi, Vahid Karamzadeh, Muthukumaran Packirisamy

**Affiliations:** Optical Bio Microsystems Laboratory, Micro-Nano-Bio Integration Center, Department of Mechanical, Industrial and Aerospace Engineering, Concordia University, Montreal, QC Canada

**Keywords:** Acoustics, Design, synthesis and processing, Polymerization mechanisms, Polymers

## Abstract

Photo- and thermo-activated reactions are dominant in Additive Manufacturing (AM) processes for polymerization or melting/deposition of polymers. However, ultrasound activated sonochemical reactions present a unique way to generate hotspots in cavitation bubbles with extraordinary high temperature and pressure along with high heating and cooling rates which are out of reach for the current AM technologies. Here, we demonstrate 3D printing of structures using acoustic cavitation produced directly by focused ultrasound which creates sonochemical reactions in highly localized cavitation regions. Complex geometries with zero to varying porosities and 280 μm feature size are printed by our method, Direct Sound Printing (DSP), in a heat curing thermoset, Poly(dimethylsiloxane) that cannot be printed directly so far by any method. Sonochemiluminescnce, high speed imaging and process characterization experiments of DSP and potential applications such as remote distance printing are presented. Our method establishes an alternative route in AM using ultrasound as the energy source.

## Abstract (web summary)

Photo- and thermo-activated polymerization and melting processes are dominant in Additive Manufacturing (AM) while ultrasound activated sonochemical reactions have not been explored for AM so far. Here, the authors demonstrate 3D printing of structures using acoustic cavitation produced directly by focused ultrasound which creates sonochemical reactions in highly localized cavitation regions.

## Introduction

Despite recent significant developments in additive manufacturing (AM) technologies, from printing materials^1–6^ to processes^7–13^, light and heat are still the only energy sources used in AM to drive chemical reactions or physical transformations of polymers. Therefore, materials in AM processes are limited to photo-sensitive resins, like in stereolithography (SLA)^14^ or direct laser writing (DLW)^15^, and thermoplastic filaments or powders, like in fused deposition modeling (FDM)^16^ or selective laser sintering (SLS)^17^. Parameters controlling the chemical interactions are defined by the amount of energy per molecule, interaction time and pressure^18–22^. Existing AM energy sources, light and heat, do not utilize all the potential of the chemistry in terms of the control parameters while sonochemistry pushes these parameters to their limits. Extraordinary high temperature (exceeding 15,000 K), high pressure (exceeding 1000 bar) and fast heating and cooling rates (over 10^12^ K/s)^18^ inside the active cavitation bubbles, known as hotspots^23,24^, surrounded by bulk liquid at room temperature, are the reasons for the sonochemical reactions.

If one could unleash the potential of sonochemistry in AM processes, an unconventional route for printing conventional as well as impossible to print materials with usual energy sources would emerge. An example for such materials is heat-curing thermoset polymers. Thermosets can be cured either optically or thermally. Additive manufacturing of optically cured thermosets is possible via light-based AM methods. However, an effective on-demand curing of heat-curing polymers are yet to be introduced due to the difficulty of applying very short heating and cooling rates at small localized regions^25^. Sonochemistry can be a solution to print such materials due to its highly localized temperature with fast heating and cooling rates. Acoustic assisted polymerization^26–28^ has been studied extensively. However, these studies were conducted in an ultrasonic bath or by a horn which lack highly focused reaction region (which is analogous to a laser beam spot in SLA or DLW) and high rate of polymerization, which are necessary for 3D printing.

**Fig. 1:**
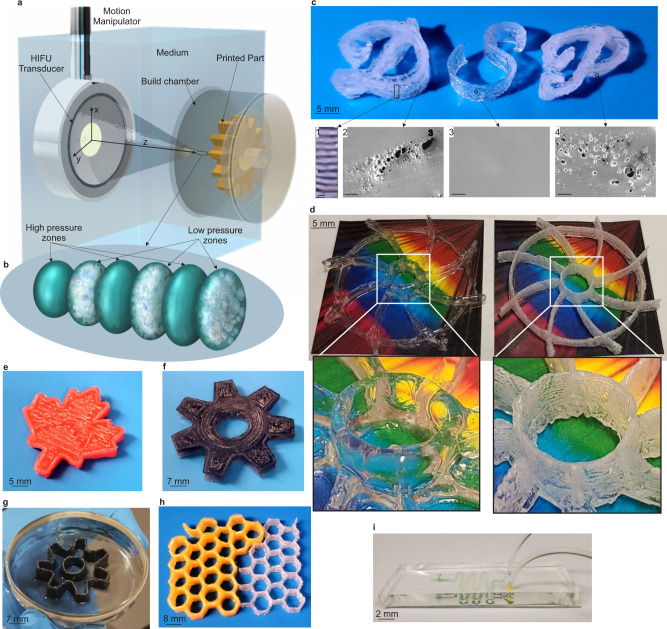
DSP concept and printed parts. **a** DSP process schematic. **b** Detailed view of UAMR, bubbles are created in low-pressure zones. **c** Printed “DSP” letters. **c1** porous/transparent structure of the printed part (the scale bar is 200 μm). **c2**–**4**, SEM pictures of the microstructure of the printed parts (the scale bar is 100 μm). **d** Printed impellers, transparent and porous. **e**–**h** Printed maple leaf, gear, shell and honey comb, respectively. **i** Molded microfluidic channel using 3D printed PDMS mold. (HIFU: high-intensity ultrasound).

Here, as shown in Fig. 1a, we 3D print objects inside a build chamber filled with the build material (monomer mixed with curing agent or different mixtures) through exposing to the focused ultrasonic field. We call this method Direct Sound Printing (DSP). The ultrasonic field, which is generated by a monolithic spherical focused transducer, reaches the build material after passing through the shell of the build chamber. At the focal location in the build material, as shown in Fig. 1a and detailed in Fig. 1b, the chemically active acoustic cavitation region solidifies the liquid resin or mixture and deposits it to the platform, or on top of previously deposited and solidified regions. We call this region as ultra-active micro reactor (UAMR) where generated bubbles and polymerized resin appear at the low-pressure zones and then they migrate momentarily to high-pressure zones until they reach to the platform or previous solidified pixel where they are deposited. The transducer is moved in the medium by a motion manipulator to locate the focal region along a calculated path in the build chamber to create the desired part pixel by pixel. Input parameters of DSP process affect the microstructure of the printed parts. These parameters are characteristics of the transducer driving pulse (such as electrical power, frequency and duty cycle which is the active fraction of the driving pulse period), build materials (such as the mixing ratio of monomer to curing agent, mixture ratio, viscosity and surface tension) and the transducer motion (such as velocity and acceleration of the transducer). Different micro structures result in optically transparent to opaque parts in DSP. The resultant opacity is due to the porous structure of the printed part, which can be controlled/by manipulating the DSP input parameters.

**Fig. 2:**
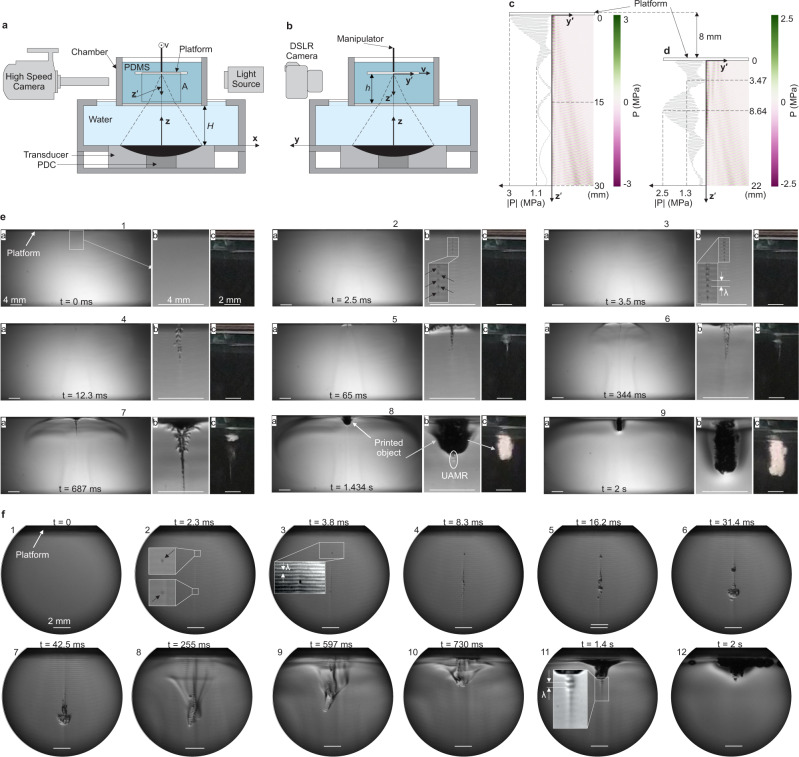
Observation of UAMR during insonication of the build chamber. **a** and **b** Front and side view of the observation setup which constitutes a high speed and DSLR camera. **c** and **d** Acoustic pressure in the build chamber, *P*, at *h* = 30 mm and *h* = 22 mm, respectively. **e** Footage from high-speed imaging (e1-a to e9-a) of area A and DSLR camera (e1-c to e9-c) for *h* = 30 mm where the acoustic focal is at the platform location (e1-b to e9-b are the magnified views of e1-a to e9-a, respectively) (DSLR: digital single-lens reflex, PCD: passive cavitation detector, UAMR: ultra-active micro reactor, PDMS: polydimethylsiloxane). **f** Footage from high speed imaging (f1-12) for h = 22 mm and v = 0. For all cases, f = 2.15 MHz, DC = 100%, and Power = 210 W.

**Fig. 3:**
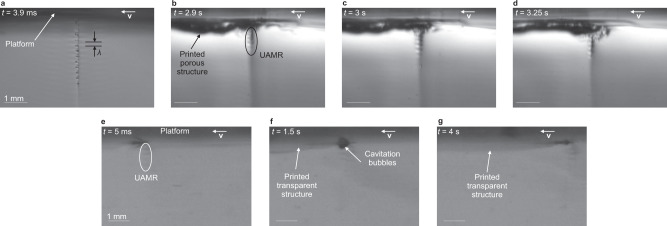
Porous and transparent printing observation of UAMR on the platform during insonication of the build chamber. **a**–**d** Porous printing progress in progressive times. *v* = 300 mm/min, and *h* = 30 mm **e**–**g** Transparent printing progress in progressive times, *v* = 240 mm/min, Power=20 W, DC = 50%.(other experimental conditions are the same as Fig. 2). (UAMR: ultra-active micro reactor).

As shown in Fig. 1c, letters of “DSP” are printed with varying mixing ratios of base Polydimethylsiloxane (PDMS) to the curing agent. Mixing ratio 10:1 (polymer base to curing agent) gives layered porous/transparent structure for letters “D” and “P” as shown Fig. 1c-1, 1c-2, and 1c-4. However, ratio 13:1 gives a transparent structure as shown Fig. 1c-3. Therefore, DPS is capable of printing parts from optically transparent to opaque (porous) parts. Although transparent PDMS is desirable in many applications, porous PDMS (sponge) has a wide range of applications^29^ too from flexible conductors to tissue engineering. Figure 1d also shows two printed parts of the same impeller geometry, one transparent and the other porous/opaque. We added dye (commercial oil color) to create colored parts as shown in Fig. 1e–h. Figure 1i shows a molded PDMS microfluidic channel using a silanized master PDMS mold printed by DSP. Supplementary Movie 1 shows the apparatus (Fig. 1a) in action while printing transparent and porous structures.

The initial idea of DSP originates in our sonochemiluminescnce (SCL) experiments on alkaline aqueous solution of luminol (3-aminophthalhydrazide) to identify the spatial distribution of active chemical regions under focused ultrasound exposure (see Supplementary Information and Supplementary Figs. 1–7). Hydroxyl radicals and hydrogen peroxide generated sonochemcially react with luminol where blue light is emitted^30–32^. We observed the localized light at the focal region at certain experimental conditions. If we could utilize this reactivity of the localized region in the printing polymer, we could drive polymerization in the region and solidify the material consequently. The resemblance of this active region to the laser beam spot SLA inspired us to seek the possibility of printing with acoustic waves. In photochemistry (as in SLA), very large amounts of energy are introduced in a short-period in the form of electrical excitation. However, in sonochemistry (as in DSP), this energy is thermal^20^ with very fast heating and cooling rate inside the cavitation bubble so that it doesn’t transfer to the surrounding liquid. This short-lived thermal energy could polymerize heat-curing polymers in DSP.

## Results

### Close observation of UAMR during insonication

In order to examine closely the concept of DSP, we conducted series of high-speed imaging tests as shown in Fig. 2 to observe the UAMR regions and the physical transformation of the liquid resin to solid in these areas. Figure 2a, b are the front and side views of the setup, respectively. PDMS is contained in the chamber in Fig. 2a, b. The chamber is placed at a constant distance, *H*, from the transducer. The glass platform is moved (along **y′** axis with velocity, **v**) and located (by a distance, *h*, from the bottom of the chamber) in the chamber by a motion manipulator. The DSP process is captured by/both high speed and digital single-lens reflex (DSLR) cameras as shown in Fig. 2a, b. The first set of tests are conducted with a static platform (*v* = 0) with *h* = 30 mm and 22 mm. 30 mm is selected because at this location the platform is placed at the highly chemically active region. The simulated linear acoustic pressure pattern for *h* = 30 mm is shown in Fig. 2c. The maximum acoustic pressure of 3 MPa is created at the platform surface where the ultrasound focal point is located. However, if the platform is lowered, the maximum pressure would not happen at the platform surface. As an example shown in Fig. 2d, if the platform is placed at *h* = 22 mm, the maximum acoustic pressure will be at 8.64 mm distance from the platform surface due to acoustic reflection from the platform. Figure 2e illustrates the captured printing phenomena in the chamber for *h* = 30 mm (corresponding to Fig. 2c). Figure 2e-1a shows the moment right before the insonication at *t* = 0 of the area A shown in Fig. 2a. Figure 2e-1b is the magnified region showed in Fig. 2e-1a. Figure 2e-1a and  2e-1c are captured by the high-speed camera and the DSLR camera, respectively, and they are timely synchronized. The cavitation bubbles start to appear at about 2.5 ms at low-pressure zones as shown in Fig. 2e-2b with arrows. The footage of high-speed imaging experiments for different *h* can be found in Supplementary Movie 2. We conducted these tests in such a way that we can be benefited by both shadowgraph technique (for observing cavitation bubbles) and Schlieren photography concept (for observing pressure waves). Owing to the acoustic pressure variation and consequent density change of the resin, low-pressure and low density regions appear lighter while high-pressure high-density regions appears darker in the high-speed images. Bubbles are generated in the low-pressure zones and then momentarily migrate to the high-pressure zones as seen in Supplementary Movies 2, 3. The distance between consecutive low or high-pressure zones is *λ* as shown in Fig. 2e-3b. *λ* = *λ*_*0*_/2 where *λ*_*0*_ is the ultrasound wavelength at the transducer driving frequency. *λ*_*0*_ = 474 μm for Sylgard-184 (as our PDMS system) medium at 2.15 MHz. The deposited area grows during 2 s of the continuous insonication and the UAMR region is clearly seen in Fig. 2e and highlighted in Fig. 2e-8b.

Figure 2f shows the footage of 2 s insonication in case of Fig. 2d where *h* = 22 mm. The high-speed imaging confirms the results of Fig. 2d in which chemically active regions are not located at the platform surface. At *t* = 2.3 ms, first bubbles appear at two locations as indicated by arrows in Fig. 2f-2. At the beginning, bubbles/solidified material moves away from the platform and against the streaming direction. The similar phenomena has been observed in boiling histotripsy of tissues that leaves a tadpole lesion area^33,34^. The opposite direction movement of the solidified material is due to the back scattering of the ultrasound field caused by the cavitation bubbles. However, after *t* = 255 ms (Fig. 2f-8), the solidified material is carried towards the platform by the acoustic streaming. When the material starts being deposited on the platform, the UAMR region appears and grows the deposition as highlighted in Fig. 2f-11.

The stationary platform was the focus of the experiments in Fig. 2. However, a dynamic test in which the platform is moved by a linear velocity, **v**, during insonication is investigated in Fig. 3. Porous (Supplementary Movie 4) and transparent (Supplementary Movie 5) lines are printed in Fig. 3a–d (mixing ratio 10:1) and Fig. 3e–g (mixing ratio 13:1), respectively. Figure 3a shows the generated cavitation bubbles at the low-pressure zones at *t* = 3.9 ms. UAMR region is observed in Fig. 3b–d, which continuously deposits material on the platform on a straight line along the platform velocity, **v**. Figure 3e–g show the printing of a transparent structure by cavitation bubbles (see Supplementary Information for discussion on porosity tuning).

The temperature at the printing spot is measured on the platform as shown in Supplementary Fig. 8a, b. The build material experiences ~8 °C temperature increase at the printing location as shown in Supplementary Fig. 8b. This amount of temperature increase alone is not sufficient at all for almost instant curing in the printing process, which shows that sonochemical reactions is critical for curing in DSP.

Acoustic streaming and velocity are shown in Supplementary Fig. 8c, d, respectively. The experiments in Fig. 3 and Supplementary Fig. 8a–d were conducted under extremely high power, 210 W, for the better understanding of the printing phenomena in DSP because the difference between high and low-pressure zones is clearer visually in the camera footage due to high induced acoustic pressures, which leads to more intense dark and light areas in the imaging. However, real printing is conducted with as low power as 20 W. The maximum streaming velocity is 20 mm/s for 210 W power, however, it is less than 3 mm/s for 20 W, which is the power used for printing transparent parts in this paper (Supplementary Movie 6). In addition, the streaming velocity on the platform where the printing happens is very small as it can be seen both Supplementary Fig. 8d, e. Therefore, although streaming happens in DSP but the printed spot doesn’t experience the high-streaming velocities. “D” and “P” letters in Fig. 1c are printed with 40 W and “S” is printed with 20 W power. As it can be seen, 20 W leads to transparent objects as 40 W leads to porous structures.

It should be noted that the equipment used in this work (transducers and pulse generator) are manufactured for the purpose of therapeutic high-intensity focused ultrasound (HIFU)^35^ applications such as tissue ablation. The acoustic intensity used in HIFU applications is greater than 1000 W/cm^2 ^^36^. However, the printing intensity at the focal location in DSP at 20 W is ~100 W/cm^2^ as shown in Supplementary Fig. 8g. The printing pressure is less than 2 MPa as shown in Supplementary Fig. 8i.

### Process and material characterization

**Fig. 4:**
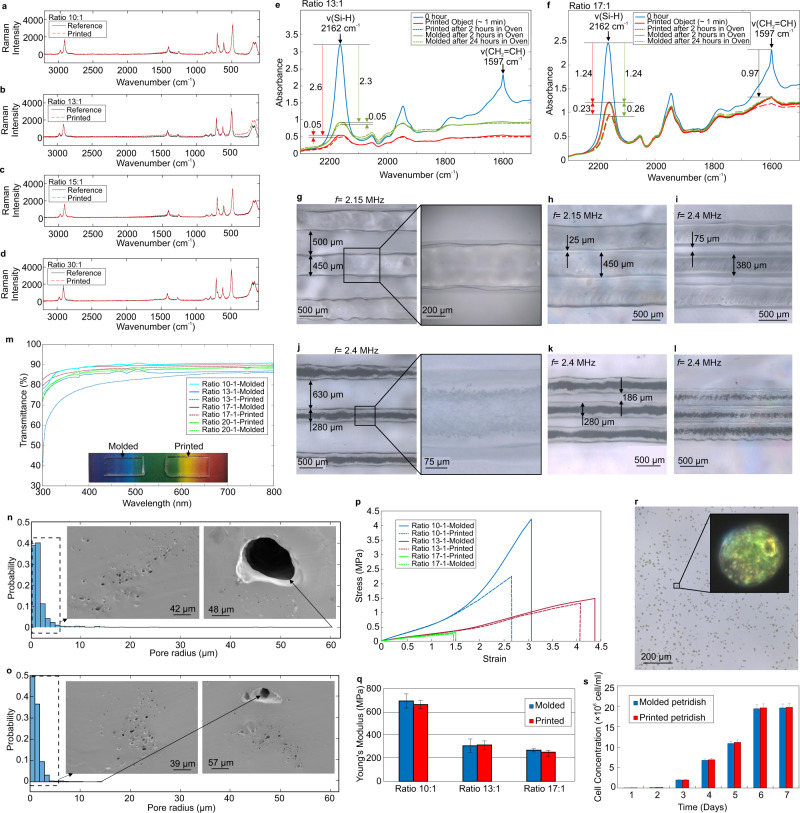
Process, material and microstructure characterization. **a**–**d** Raman spectrums of reference (molded) and printed PDMS for different mixing ratios. **e** and **f** IR bands comparison of Si-H (2162 cm^−1^) and vinyl (1597 cm^−1^) stretches for maxing ratio 13:1 and 17:1, respectively. **g**–**i** XY resolution investigation for mixing ratio 15:1. **j**–**l** XY resolution investigation for mixing ratio 10:1. **m** UV–Vis spectrum comparison among printed and molded specimen for different mixing ratios. **n** and **o** Samples of pore size distributions for printed objects in 10:1 ratio with frequencies 2 MHz and 3.1 MHz, respectively. Transducer H-316 (natural frequency: 2.5 MHz) is used for printing. **p** and **q** Stress–strain curves and Young’s Modulus comparison, respectively, of printed and molded specimen for different mixing ratios. **r** Microscopic picture of a Chlamydomonas reinhardtii at 7th day of cell culture of a printed petridish. **s** Cell concentration of Chlamydomonas reinhardtii after every day for 7 days for the printed and molded petridishes.

Material characteristics of normally cured PDMS samples in room temperature are compared with printed walls using DSP method for different PDMS mixing ratios by Raman Spectroscopy as shown in Fig. 4a–d. Raman spectrum of printed materials are in good agreement with the normally cured PDMS (reference spectrum in Fig. 4a–d). This shows that DSP technology results in the same material as the normally cured PDMS.

The PDMS system used in this paper is Sylgard 184, which is a two component system. The elastomer and the curing agent contain vinyl-terminated siloxane and Si-H moieties, respectively. The crosslinking happens between vinyl group and Si–H bond^37^. During curing, the intensity of characteristic infrared (IR) bands of vinyl and silane (Si-H) stretches reduce; these reductions can be used as an indication of the curing progression or curing rate^25,37^. The Si-H intensity change is far greater than vinyl group, which makes the silane bond intensity more sensitive indication of the course of reaction. We measured IR bands of PDMS systems (Fig. 4e, f): mixture of base and curing agent, printed PDMS via DSP and molded PDMS in oven at 40 °C. For mixing ratio 13:1 (Fig. 4e), molded PDMS in oven (at 40 °C for 2 h, which leads to a solid part than can be removed from the mold easily) shows 72% loss in Si-H intensity where we observed 84% reduction in intensity in the case of printed PDMS (printing took less than a minute to print a test sample wall as that of molded specimen for the IR measurements that took 2 h in oven at 40 °C). For mixing ratio 17:1 (Fig. 4f), we observed 52% Si-H intensity reduction in both molded (in oven at 40 °C for 2 h) and printed cases. Keeping both the molded and printed parts further in oven did not reduce the intensity of Si-H significantly. The IR measurements of mixing ratio of 20:1 is shown in Supplementary Fig. 9a, which show 77% reduction in Si-H intensity of the printed part (similar to one day in the oven for the molded part). Our IR experiments show that the curing rate or the curing agent consumption in the printing process of DSP is similar to the molded parts placed in oven at 40 °C for a day.

The XY printing resolution in DSP depends on the input parameters of the process of which ultrasound frequency plays a key role, higher the frequency smaller the feature size. We printed sets of three parallel lines with different gaps (see Supplementary Fig. 10a, b). We reduced the gap until the lines are not distinguishable from the gap (the gap is filled). 2.15 MHz ultrasound frequency leads to 450 μm lines with 500 μm empty gaps as shown in Fig. 4g (since the PDMS mixing ratio is 17:1 in this experiment the printed part is transparent). We could reach to 25 μm gap width with 450 μm lines as shown in Fig. 4h with 2.15 MHz. However, in order to increase the gap width, we no longer could use 2.15 MHz because the lines were connected. Instead, we used 2.4 MHz ultrasound frequency (0.25 MHz increase), which leads to smaller line width of 380 μm and larger gap width of 75 μm (Fig. 4i).

Printing with the 2.4 MHz in printing material with mixing ratio of 10:1, which gives porous structures, leads to 280 μm line and 630 μm gap (Fig. 4j). We could decrease the gap to 186 μm with 280 μm under 2.4 MHz (Fig. 4k). However, decreasing the distance between the lines to 300 μm closed the gaps between the lines (Fig. 4l). Figure 4g–l are captured by Confocal Laser Scanning Microscope. Same pictures are captured under a normal laboratory microscope in Supplementary Fig. 10c–e.

The geometry of the spherically focused transducer also affects the printing resolution since the focal size depends on it. The focal spot size considering −6 dB drop off points, *w*_d_, depends on geometrical parameters such as the radius of the curvature of the transducer surface, *RT*_*0*_, and transducer exit plane radius (active radius), *a*, and can be represented^38^ approximately as *w*_d_ = 4.45*RT*_*0*_/(*ka*), where *k* is the wave number. *w*_d_ decreases if *RT*_*0*_/*a* decreases. Smaller focal size means smaller printing spot and finer resolution. In addition, the effects of hydrostatic pressure and temperature during printing are investigated in Supplementary Information and Supplementary Figs. 11 and 12.

We could print transparent and porous (opaque) structures by tuning the mixing ratio of the base and the curing agent of PDMS. Keeping the ultrasound power constant, mixing ratios less than 13:1 led to porous structures and ratios higher than 13:1 led to transparent structures. Increasing the power (more than 30 W) tends to increase the porosity and utilizing less power helps (less than 30 W) printing more transparent parts. The transparency evaluation has been conducted by UV–Vis spectrometry of printed and molded 3 mm thick walls (Fig. 4m) for ratios of 13:1, 17:1 and 20:1. Transmittance of printed and molded parts of 17:1 and 20:1 are in good agreement for all wavelengths even for non-visible regions. The printed 13:1, in comparison with molded 13:1, showed ~20% and ~10% less transmittance for wavelengths less than 400 nm and between 400 nm and 500 nm, respectively. However, the transmittance difference falls less than 5% for wavelengths larger than 500 nm. The reason for the less transparency in the ratio 13:1 could have roots in the fact that ratio 13:1 is the border ratio between visible transparent and porous structures and therefore there could be still some submicron pores in the structures, however, not visible. We expect that with further investigation of power and frequency, other ratios less than 13:1 could also be printed transparently by DSP.

The DSP input printing parameters could affect the microstructure of the printed parts. Lower frequency leads to larger feature size and larger range of pore size in comparison with higher frequency, which leads to smaller feature size and narrow range of pore size. Figure 4n, o show the pore size distribution of the cross section of the printed part with 10:1 mixing ratio for 2 MHz and 3.1 MHz. As it can be seen, sample Scanning Electron Microscope (SEM) pictures corresponding to the pore size distribution are shown as well. Pore sizes are the results of the cavitation bubbles, which their distribution can be estimated by bubble dynamics (see Supplementary Information). Supplementary Figs. 13 and 14 provide further results of porosity analysis and also bubble dynamics for each printing condition.

We conducted classical tensile stress–strain measurements (see Methods and Supplementary Fig. 15) to investigate the mechanical properties of the printed parts and compared them to molded parts with different ratios (Fig. 4p, q). Young’s Modulus of printed and molded parts are in good agreement (Fig. 4q) for all ratios. The elongation at break for ratio 10:1 for printed part is ~15% less than molded one due to its porous structure. Ratio 13:1 led to less pores than 10:1 and closer elongation at break in comparison with the molded parts are expected. The tensile test proves it as the elongation at break for printed part with ratio 13:1 is ~7% less than the molded part. The stress–strain curve of the printed and molded parts with 17:1 are in good agreement even at the break.

Since we printed Sylgard-184 directly and purely without adding any toxic additives, the biocompatibility of the printed parts are expected. However, we investigated the biocompatibility of our printed parts and compared it with molded Sylgard-184 through cell culture of Chlamydomonas reinhardtii^39^, green algae, in printed and molded petridishes (see Methods and Supplementary Fig. 16). We followed the cell count for the course of 7 days. The cell concentration after 6th day (Fig. 4r shows the cells and detailed view of a cell at day 6) plateaued for both printed and molded petridishes. Cell concentration are in good agreement between printed and molded petridishes (Fig. 4s), which proves that the biocompatibility of the printed parts in DSP is as the same as the molded parts.

### Potential applications of DSP

In this section, a few potential applications of DSP covering 3D printing of polymers, metal and ceramic composites, inside body bioprinting, multi functional plasmonic integrated platform for biosensing and PDMS microfluidic chips are introduced and demonstrated. However, DSP is first and foremost an AM method to print materials that were difficult to print by existing AM methods. One example of such materials is heat-curing thermosets and Sylgard-184 (as a system of PDMS) is one of the most studied of such thermosets in both porous and non-porous (transparent) forms. Porous (sponge) PDMS^29^ has a wide range of application such absorbents and oil/water separation^40^, flexible conductors^41^ and energy harvesting and storage devices^42^ while the application of the transparent PDMS includes: lab-on-chip^43,44^, tissue and organ-on-a chip devices^45–47^ and biological machines^48,49^ due to its biocompatibility, transparency and gas permeability properties^50,51^. Sophisticated light-based AM is able to print optically cured silicone elastomers^52–54^. However, this method requires postprocessing of some of the printed parts to remove remaining toxic photopolymerization byproducts and unreacted compounds by solvents^52^. Moreover, there is no practical solution for printing thermally cured thermosets^25^, in this case heat-curing PDMS, as always an additive^55^ needed to be introduced to change the rheology of the printing material^56^, mainly to increase the viscosity of the deposited material to stand long enough for heat-curing or release the material completely in a supportive bath^57–59^. Adding another part to the PDMS system (polymer base and curing agent) changes the material properties and involving a filling structure or the bath could create geometric inaccuracies due to the weight of the PDMS and the post-processing is needed to remove the support bath material^57^. Plasmonic heating of heat-curing PDMS by adding gold nano particles^25^ showed that the resultant plasmonic nanosecond heating and cooling cycles could increase the curing rate and on-demand curing could be possible. However, AM applications of gold nano particles are yet to be proved practically and moreover, gold is expensive and it needs to be extracted after printing, which proven to be difficult^60^. Here, we showed that DSP is capable of printing this material directly without any additives, support bath or material engineering while keeping Sylgard-184 material properties. In addition to Sylagrd-184, we could DSP print successfully in different industrially available heat-curing thermosets and elastomers such as DOWSIL™ EE-1010, DOWSIL™ EE-3200, DOWSIL™ EI-1184, SYLGARD™ 182 and SYLGARD™ 186. We expect DSP finds more material candidates primarily from other heat-curing thermosets with low-exothermic characteristic to ensure proper printing resolution.

**Fig. 5:**
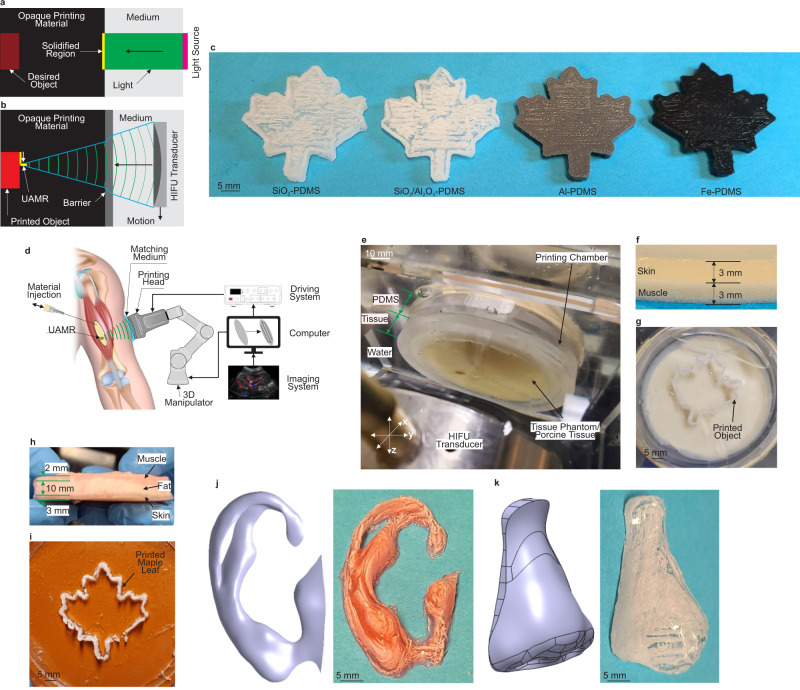
Potential applications of DSP. **a** Light-based AM and small cure depth for opaque printing materials. **b** DSP and deep penetration of ultrasound in opaque printing materials. **c** Printing opaque micro/nano composites by DSP. **d** The concept of an ideal DSP technology for noninvasive inside body printing. **e** in-vitro/ex-vivo prove of concept setup. **f** Tissue phantom of human skin and muscle. **g** A printed maple leaf using the tissue phantom. **h** Real porcine tissue compromising skin, fat and muscle. **i** Printed maple leaf using the porcine tissue. **j** and **k** Printed ear and nose using the tissue phantom. (HIFU: high-intensity focused ultrasound, UAMR: ultra-active micro reactor, PDMS: polydimethylsiloxane).

Another distinctive aspect of DSP is the more depth of penetration of the printing acoustic energy. In conventional light-based AM technologies, light absorption and scattering hinder large penetration of the printing energy in the printing medium as shown in Fig. 5a especially if the material is opaque and filled with scattering particles. However, the optical opacity of the printing material does not affect the DSP process since the sound waves are used as the energy source and not light as shown in Fig. 5b. Acoustic attenuation and scattering that diverge sound waves need to be taken into account based on the printing materials. However, due to the nature of sound waves, light absorption and scattering do not affect the depth of sound wave penetration in optically opaque materials as long as acoustic attenuation and scattering are not dominant. Figure 1e–h show the parts that are printed out of completely opaque printing materials where the ultrasound waves traveled through 30 mm of water and 1 mm of a solid barrier and 18 mm of the opaque printing material to reach the platform and start printing on it. Opaque micro/nano-composite materials are printed in the same manner as shown in Fig. 5c (Supplementary Movies 7 and 8). Colloidal solutions of the opaque printing materials are prepared by mixing silica, silica/alumina, aluminum and iron powders with the polymer matrix (see Methods). The printed parts are used as green parts for making ceramic composite objects obtained after sintering in a furnace (see Methods and Supplementary Fig. 17a). The distance between the energy source and the printing location where the ultrasound passes through obstacles in between introduces a concept of Remote Distance Printing (RDP) in which objects can be created remotely without direct access to the printing spot. RDP could have many applications in different disciplines such as remote repairing or on-site maintenance of hidden parts in aerospace industries and in-vivo remote and noninvasive bioprinting of inside body parts in medical applications. In the following, the medical application is investigated.

The importance of combination of printing technologies and injectable biomaterial for the purpose of inside body printing has been introduced 2 decades ago^61^. However, most of the cutting edge bioprinting technologies of today require open surgeries^62,63^ due to the fundamental limitation of the current printing methods that use heat or light as the energy source. Recently, nearinfrared (NIR) source was used to print structures noninvasively under skin (0.5 mm thick)^64^. However, this small penetration makes the bioprinting limited to submillimeter depths. The nature of DSP enables creating structures deep inside body in the range of few dozens of millimeter. The schematic view of an ideal DSP technology for noninvasive surgery is illustrated in Fig. 5d where printing process is integrated with an imaging system. We proved this concept by our in-vitro/ex-vivo setup shown in Fig. 5e. In our in-vitro experiment, tissue mimicking phantoms of human skin and muscle are created (see Methods for the preparation of the tissue phantoms) and shown in Fig. 5f. Then we printed structures where ultrasound passes through water (matching medium in Fig. 5d) and 6 mm tissue phantom and 18 mm PDMS (the total printing depth is 24 mm). Figure 5g shows the printed object attached to the substrate in the printing chamber. In our ex-vivo experiment, we used the real porcine tissues as shown in Fig. 5h. In this experiment (Supplementary Movie 9), focused ultrasound passes through 15 mm tissue (skin, fat and muscle) and 18 mm PDMS (the total printing depth is 33 mm) and the printed object is shown in Fig. 5i. More complex geometries such as an ear and nose are printed in our in-vitro setup (Supplementary Movie 10) as shown in Fig. 5j, k, respectively. Therefore, DSP introduces the possibility of noninvasive deep inside body printing.

In another application, DSP can be used for selective integration of functionalities such as electrical, optical, etc. to desired objects as we demonstrate (see Supplementary Information, Supplementary Figs. 18 and 19 and Supplementary Movies 11–13) simultaneous synthesis and patterning of nano particles due to localized chemical activity of UAMR leading localized and selective patterning and making gold nano particle-PDMS composite.

Although the mechanism for introducing DSP technology in this paper (Fig. 1a) is very general and has some unique aspects such as RDP, it has some limitations in the complexity of the printed parts. It is difficult to perform multi-material printing with the mechanism in Fig. 1a since the build chamber should be refilled each time with a new material that is going to be printed. Another difficulty is printing overhanging structures by the mechanism in Fig. 1a, since the printed spot needs to be supported. An alternative mechanism based on the same DSP concept along with the use of support materials can help resolving these problems. This is the topic of the future research of the authors. We applied DSP up to the volumetric deposition rate of ~15,000 mm^3^/h, which is comparable with FDM and Direct Ink Writing (DIW)^9^. A schematic holistic comparison of the volumetric deposition rate vs resolution between light and heat-based AM and DSP are shown in Supplementary Fig. 20.

## Discussion

We introduce an alternative printing method, DSP, to 3D print objects using sound waves. Acoustic cavitation creates chemically active regions in the printing resin or resin mixture medium in which the resin undergoes fast phase transformation from liquid to solid under sonochemical reactions. With DSP, heat-curing polymers with free radical polymerization process such as heat-curing thermosets, which could not be printed directly by photo or heat energy sources in AM processes, can now be printed directly. The basic mechanism of DSP is presented using SCL experiments. The chemically active 3D printing region in DSP is analogous to a laser beam spot in common photo or heat-based AM technologies. Our high-speed imaging experiments show the presence of UAMR regions in which the active cavitation bubbles are created and chemical reactions responsible for phase transformation of the material occurred. Material characterization tests show no difference between normally cured PDMS system and printed one by DSP. Printed parts have porosity size range which depends on the printing input parameters such as printing material, ultrasound power, frequency, duty cycle and transducer velocity. To show unique future potentials of DSP, applications such as RDP for inside body bioprinting and direct nano particle synthesizing and pattering by DSP for integrating localized surface plasmon resonance with microfluidics chip are experimentally demonstrated. Cavitation phenomena, which is known to be the cause of destruction, is a force of creation in DSP. Our method tames cavitation by harnessing one of the oldest energy sources known, sound waves, to create physical objects.

## Methods

### Material preparation

Luminol solution for SCL experiments: 1 mM luminol (3-aminophthalhydrazide, Sigma-Aldrich, Canada) solution is prepared and pH 12 is obtained by adding NaOH (Sigma-Aldrich, Canada) and measuring pH by a pH 315i meter (Wissenschaftlich-Technische Werkstätten, WTW, gmbh, Germany) in real time. Then, 0.5 M sodium carbonate (Na_2_CO_3_) is dissolved in the solution. Sodium carbonate is prepared by heating sodium bicarbonate (NaHCO_3_, baking soda) at 100 °C. PDMS for 3D printing tests: SYLGARD 184, polydimethylsiloxane, kit (Krayden, Canada) is used. The PDMS monomer and curing agent were mixed with the desired mixing ratios. The mixture was degassed using a vacuum for 45 min. Colored PDMS for 3D printing tests: The PDMS color was modified by adding the 2% w/w oil-based dye (Winsor & Newton, UK) to the base monomer before mixing with curing agent.

### Transducers, pulse generator, passive cavitation detector, and needle hydrophone

Two HIFU transducers are used in this paper, H-148 and H-316 (Sonic Concepts Inc., USA). Specifications of H-148 and H-316 specifications are as fundamental frequency, 2.15 MHz and 2.5 MHz, minimum frequency, 1.46 MHz and 2.06 MHz, maximum frequency, 3.2 MHz and 3.66 MHz, active diameter, 64 mm and 72 mm, radius of curvature, 63.2 mm and 50 mm, focal depth from exit plane of the transducer housing, 51.54 mm and 30.33 mm, −6 dB focal size at the fundamental frequencies, 0.7 × 5.33 mm and 0.43 × 2.29 mm, respectively. Efficiency of acoustic power from electrical power is 85% for both transducers. H-148 has a 22.6 mm diameter central hole in which a passive cavitation detector (PCD), Y-107 (Sonic Concepts Inc., USA), is mounted. The transducer is driven using the transducer power output device TPO-102 (Sonic Concepts Inc., USA), which provides the sinusoidal output powers in a range of 5 to 210 Watts. A 0.2 mm dia. needle hydrophone, model no. D1602 with hydrophone booster amplifier (Precision Acoustics Ltd., UK), is used for the temporal pressure measurement at a location very close to the focal region (based on the manufacturer’s instruction) in water at the back of the build chamber (Supplementary Fig. 8f).

### SCL experiments

The setup of SCL experiments (Supplementary Fig. 1a, d, and g) constitutes the transducer, H-148, and PCD, Y-107, which are mounted in the bottom of the plexiglass container, the DSLR camera EOS 500D (Canon Inc., Japan), a glass platform which is connected to a computer numerical controlled, CNC, machine (Stepcraft Inc., USA). The entire setup is placed in a dark room. In Supplementary Fig. 1a, d, the plexiglass container is filled with the luminol solution. In Supplementary Fig. 1g, the plexiglass container is filled with degassed deionized water and a polystyrene container is filled with the luminol solution. The images were captured by the DSLR camera with ISO 3200 and exposure time of 10 s or 30 s. PCD data is collected by the tektronix DPO 2024B oscilloscope (Tektronix, USA).

### Close observation experiments and temperature measurements

The Phantom v2012 (Vision Research Inc., USA) is used as the high-speed camera for high-speed imaging and particle tracing experiments. Nikon SWM VR IF Micro 1:1 lens is used for capturing pictures in Fig. 2e. Optem Fusion 7:1 lens setup is used for Figs. 2f and  3. Halogen light source of variable power is used for all the experiments. To quantify the temperature gradient during the printing process, the tip of a 0.125 mm Copper-Constantan T-type thermocouple (Omega Engineering Inc., USA) with the maximum permitted error of 0.5 °C is inserted in the printing platform at the center of a 6 cm straight path. A through all hole is drilled in the platform and the thermocouple is inserted through this hole in such a way that the tip of the thermocouple has a 0.2 mm stick out from the surface of the platform where the printing happens. Therefore, due to the stick out, which makes the tip to be part of the final printed line, the measured temperature is the in-process temperature of DSP. Temperature data is acquired by DAQ NI 9212 (National Instrument Co., USA) with the sample rate at 95 Samples/s and the temperature measurement sensitivity of 0.01 °C, which is coupled with the sensor. The real time temperature is collected as shown in Supplementary Fig. 8b.

### Postprocessing in DSP

The printed parts are washed with isopropanol alcohol (IPA) to remove the uncured materials (similar to SLA printed parts) from the surface of the printed parts. Then the parts are placed in a 40 °C oven for half an hour for drying the washed parts and complete the curing process.

### UV–Vis measurements

Transmittance spectra of the specimens for UV–Vis experiments the samples was collected on a LAMBDA 650 UV–Vis spectrophotometer (PerkinElmer Inc., USA).

### Tensile tests

The standard tensile tests were carried out on the Type V specimens using motorized test stand (Z5, Hoskin Scientific LTD.) equipped with a 1000 N load cell according to the ASTM D638. The tests were performed at a displacement rate of 10 mm/min. The force-displacement data were recorded by the dedicated software. The slope of the initial linear segment of the stress–strain curve was used to calculate the Young’s modulus.

### FT-IR measurements of the curing progression

The absorbance measurements of FT-IR specimens were carried out on a PerkinElmer Spectrum 400 FT-IR/FT-NIR spectrometer with a liquid transmission cell attachment (KBr windows, 1 mm path length), as shown in Supplementary Fig. 9b, c.

### Cell culture

Six petridishes (Supplementary Fig. 16a), three 3D printed and three molded, were fabricated. All cavities were made of Sylgard 184 with a ratio of 15:1. Tris Acetate Phosphate (TAP) media was prepared to grow the algal cells by adding the ammonium chloride, magnesium sulfate hexahydrate, and calcium chloride dehydrate to the deionized water. After preparing the TAP media, the media was autoclaved at 121 °C for 30–45 min. Then, to prepare a culture, a pinch of Chlamydomonas reinhardtii (strain- CC-125, chlamy.org, Algal Resource Center, University of Minnesota, USA) was transferred to the Erlenmeyer flask containing 50 ml TAP media using a sterilized needle. To prevent contamination of the prepared media, the Erlenmeyer flask was covered with industrial aluminum wraps and was placed on the orbital shaker (Supplementary Fig. 16b) at 100 rpm. After an hour, each circular container was filled with 1 ml of the culture media collected from the Erlenmeyer flask. All containers were placed in a 6-well culture dish and capped to prevent further contamination. Then, the culture dish was placed on the orbital shaker at 100 rpm besides the buffer Erlenmeyer flask. The cultures were grown at room temperature of 23 °C in the presence of fluorescent illumination (≈5000 lux). The growth dynamics of the algal culture in all cavities were investigated every 24 h for seven consecutive days by counting the number of algae cells under the microscope. For each observation, 10 µl drop of the culture media was collected, placed on the slide, and covered with the coverslip.

### SEM pictures of printed wall cross section

In order to investigate the porosity size of the printed parts, multiple walls with 60 mm length and 5 mm height with different printing conditions are printed. After printing, each wall is washed and rinsed with IPA to remove the unpolymerized PDMS residual. Three samples are printed using the same printing conditions and each sample is cut in three locations perpendicular to the length of the wall (the sample). A thin film is extracted from each cross section. Prepared samples are rinsed in IPA and fixed over the surface of the sample holder. Prior to imaging, samples are coated with 60 nm of the gold layer. All images are captured using the Hitachi SEM S-3400N (Hitachi, Japan). SEM pictures are analyzed using a MATLAB code^65^ and the porosity sizes of each cross section are calculated. Porosity distribution and sample SEM pictures are shown in Fig. 4n, o and Supplementary Figs. 13 and 14 for each printing condition.

### Potential applications of DSP (preparation of colloidal solutions as opaque printing materials and pyrolysis of printed parts)

7-wt% silica and silica/alumina micro particles (AEROSIL® OX50, COK 84, respectively, Evonik Co., US) are mixed with 84.5-wt% PDMS base-material by a homogenizer (Omni Mixer Homogenizer, Omni International, US) for 1 h and then 8.5-wt% curing agent is added and mixed for 15 min. 7-wt% of Iron and aluminum (99.9%, 800 nm, US Research Nanomaterials, Inc., US) is mixed with 8.5-wt% curing agent and ultrasonicated (VCX-500, Sonics, US) for 10 min. Then the prepared colloidal solution is mixed with 84.5-wt% PDMS base-material for 30 min. The printed parts are used for prepolymer ceramic green parts and are sintered in 1100 °C in a tube furnace under argon gas flow with heating and cooling rates of 40 °C/h. The resulted sintered ceramic parts are shown in Supplementary Fig. 17a. Supplementary Fig. 17b shows the X-Ray diffraction (XRD) analysis of the ceramic parts scaled to the background. The measurements are done by an Empyrean system using Cupper radiation in Bragg-Brentano reflection geometry. The ceramic parts are amorphous with very broad peaks with a few weak sharper peaks. SiC, Si, Al_2_O_3_ and 3Al_2_O_3_.2SiO_2_ are identified for the Al-PDMS ceramic part from crystalline peaks as shown in Supplementary Fig. 17c. Fe_3_Si, Fe_2_ and Fe_5_Si_3_ are also identified from XRD data for the Fe-PDMS ceramic part. Fourier-transform infrared spectroscopy (FT-IR) of Fe-PDMS before and after pyrolysis are shown in Supplementary Fig. 17d. Assignments of infrared (IR) bands in the spectrum of the Fe-PDMS sample (the printed part) before pyrolysis are as follows: 2950 and 2820 cm^−1^ (γ(CH_3_), γ(CH_2_), stretching vibrations), 1400 cm^−1^ (δ_symm_ Si-CH_3_, in-plane symmetrical deformation vibration), 1250 cm^−1^ (δ_asymm_ Si-CH_3_, in-plane asymmetrical deformation vibration), 1080 and 1015 cm^−1^ (γ_Si-O-Si_, stretching vibration), 860 cm^−1^ (Si-CH_3_, out-of plane deformation (rocking) vibration) and 790 cm^−1^ (γ_Si-C_ stretching vibration). Assignments of Fe-PDMS after pyrolysis are as: 1100 and 1080 cm^−1^ (γ_Si-O_ in Si-O-Si groups) and 770 cm^−1^ (γ_Si-C_ stretching vibration). The C-H absorption band is visible in the before pyrolysis sample, where it is not detected after pyrolysis at 2970 cm^−1^. Both before and after pyrolysis, absorption peaks are visible around 1000 cm^−1^ where siloxanes have strong bands. No changes in the intensities of the Si-O and Si-C bands, due to the presence of Fe, have been observed in the spectrum.

### Preparation of tissue phantoms

The prepared tissue phantom was composed of two bonded layers, muscle and skin. The tissue phantom of the skin comprises three layers that mimic the epidermis, dermis and hypodermis layers of a real skin tissue. The hypodermis solution is prepared^66^ by dissolving 2 %w/v gelatin (Type A procine powder, 300 g Bloom, Sigma-Aldrich Co., Canada), 0.2 %w/v agar (A1296 powder, Sigma-Aldrich, Canada) in 80 ml distilled water along with gradually adding 15 %w/v bovine serum albumin (BSA) (≥95% purity, Sigma-Aldrich, Canada), and finally mixing with 1 %w/v of silica (AEROSIL® OX50, Evonik Co., US). Similarly, the dermis mimicking solution is made from a mixture of 1 % w/v agar, 24 %w/v gelatin, 35 %w/v BSA, and 0.5 %w/v silica microspheres in 80 ml distilled water. The epidermis solution is prepared by mixing 5 %w/v glycerol, 10 %w/v gelatin powder and 0.1 %v/v glutaraldehyde (Grade I, Sigma-Aldrich, Canada). The 50 ml solution for fabrication of the muscle tissue phantom is made by mixing 40%v/v evaporated milk, 2%w/v silica powder, 2% w/v agar and 60%v/v distilled water^67^.

### Protocol of synthesizing and pattering of Gold nano islands in biosensing micro-chip experiment

Gold(III) chloride trihydrate (HAuCl_4_.3H_2_O) (Sigma-Aldrich, Canada) (.2 % w/v) in DI water is used for synthesizing and patterning using DSP. Exosomes are captured based on an affinity-based approach^68^. The biosensing protocol is illustrated in Supplementary Fig. 18. The absorption spectrum of the collection chamber is measured by an Ocean Optics USB4000 spectrometer (Ocean Insight, US) after every stage of the following protocol. Initially, the absorption spectrum of the AuNIs (Supplementary Fig. 18a) is measured. Next, a Nano Think 11 solution is passed through the channels of the chip at the flow rate of 10 μl/min for 30 min. The chip is incubated for 3 h for creation of hydroxyl bonds (Supplementary Fig. 18b). The spectrum is measured. N-(3-Dimethylaminopropyl)-N′-ethylcarbodiimide hydrochloride (EDC) and N-Hydroxysuccinimide (NHS) (Sigma-Aldrich, Canada) mixture is infused at 10 μl/min. The chip is incubated for amidation for 4 h (Supplementary Fig. 18c). The procedure is repeated (Supplementary Fig. 18d–f) for the streptavidin (0.19 nM) (IBA GmBH, Germany), biotin-PEG-Vn96 (0.87 nM) and the MCF7 exosomes (supplied by the Atlantic Cancer Research Institute (ACRI) in NB, Canada). Gold Localized Surface Plasmon Resonance (Au LSPR) is proportional to the exosome concentration.

## Supplementary information

Supplementary Information

Peer Review File

Description of Additional Supplementary Files

Supplementary Movie 1

Supplementary Movie 2

Supplementary Movie 3

Supplementary Movie 4

Supplementary Movie 5

Supplementary Movie 6

Supplementary Movie 7

Supplementary Movie 8

Supplementary Movie 9

Supplementary Movie 10

Supplementary Movie 11

Supplementary Movie 12

Supplementary Movie 13

## Data Availability

The data that support the findings of this study are available from the corresponding author upon request.

## Code availability

The codes used are open source (HITU_Simulator v2.0 and COMSOL Multiphysics 5.4) and MATLAB codes will be available upon request.
