# Supplementary material for: Direct sound printing
Source: Nat Commun. 2022 Apr 6;13:1800. doi: 10.1038/s41467-022-29395-1 (PMC8986813; doi:10.1038/s41467-022-29395-1)
Supplement: Supplementary file 1 — Supplementary Information [file 41467_2022_29395_MOESM1_ESM.pdf]

Supplementary Information

Direct Sound Printing

Authors: Mohsen Habibi[1], Shervin Foroughi[1], Vahid Karamzadeh[1], Muthukumaran Packirisamy[1*]

Affiliations: [1]Optical Bio Microsystems Laboratory, Micro-Nano-Bio Integration Center, Department of Mechanical, Industrial and Aerospace Engineering, Concordia University, Montreal, QC, Canada.

* **Corresponding author**: Email: pmuthu@alcor.concordia.ca

1. The origin of DSP in SCL experiments

The SCL experimental setups are shown schematically in Supplementary Figs. 1a, d and g. In all setups, SCL patterns, captured using a digital single-lens reflex (DSLR) camera, constitute general five regions, I to V. Region I is the most focused reactive location which resembles the laser beam spot in SLA. Ultrasound is transmitted directly to the luminol solution in Supplementary Fig. 2a. We captured the SCL patterns for varying electrical power (Supplementary Fig. 2) and duty cycle, DC (Supplementary Fig. 3). Electrical pulse period is set to 2.5 ms in the present paper therefore DC 100% and 30% means 2.5 ms and 0.75 ms active pulse duration, respectively. Supplementary Figs. 2b and c show axial SCL distribution spectrum (ASDS) diagrams, which are the normalized color intensity (*CI*) of the captured pictures along z-axis, for varying power and DC, respectively, corresponding to Supplementary Figs. 2 and 3. Supplementary Figs. 1b and c show that in wide range of powers and DCs, region I remains chemically active as *CI* level indicates. If we could harness the reactivity of this region to drive radical polymerization via sonochemical route, this region would solidify the liquid printing material locally. However, the solidified material would move due to acoustic streaming along streamlines and consequently nothing would remain at the desired printing locations (pixels of the desired part). If the solidified material is made to stick to a platform, then this problem would be resolved as the streaming forces will be less than adhesive or bonding strength. We conducted tests with the presence of a platform with varying height, *h*, (Supplementary Fig. 1d) to investigate any change in the location of region I in ASDS diagrams. ASDS diagrams for power 210 W & DC 100% (Supplementary Fig. 4) and power 210 W & DC 30% (Supplementary Fig. 5) are shown in Supplementary Figs. 1e and f, respectively. Reducing the platform height disturbs the SCL patterns, however, region I remains present and active until the platform height is less than ~ 50 mm (the geometric focal is located at 54.5 mm with respect to the transducer face). The luminol solution is contained in a chamber, as shown in Supplementary Fig. 1g, to investigate the effect of ultrasound transmission from degassed deionized water through a barrier (the chamber shell) on the ASDS diagrams for varying *h*. Figs. 2h and i show that despite compaction of the five SCL active regions in the chamber, region I still could be identified. Supplementary Figs. 6 and 7 show the SCL patterns for this setup. The idea of DSP method emerges from the observation of these

tests and taking advantage of presence of region I which is localized and chemically reactive as well as useful for printing. If we could harness the reactivity the region I on the platform (the platform is kept at region I) in the chamber filled with the resin, a 3D object could be created pixel by pixel by moving the transducer or the platform along a designated path.

2. Tuning microstructure between transparent and porous structure in DSP

The viscosities of the base and the curing agent are 5100 cP[1] and 110 cP[2], respectively. The viscosity of the mixture of 10:1 (10 base and 1 curing agent) is 3500 cP[1]. The curing agent has lower viscosity and adding more curing agent, assuming keeping the base weight constant, leads to less viscous mixture and vise versa. For example, 13:1 mixture is more viscous than 10:1 mixture. In DSP, mixing ratio 13:1 for Sylgard 184 works as a borderline ratio, higher than 13:1 (14:1, 15:1 and so on) results in transparent structure while ratios lower than 13:1 (12:1, 11:1 and so on) lead to porous structures.

The reason behind this phenomenon can be explained by the bubble rupture at the collapse phase. The chemically active bubbles undergo collapse phase (the chemically active phase of bubble oscillation in the acoustic field when temperature and pressure inside bubble reach their maximum). The cavitation bubbles which undergo collapse are also called inertial cavitation (IC) bubbles. The word "inertial" refers to the fact that the inertia of the surrounding liquid flows toward a bubble during the bubble collapse[3]. The IC bubbles upon rupture due to collapse create chemically inactive bubbles called "daughter bubbles"[4] which are then dissolved in the medium. In DSP, created IC bubbles undergo collapse. During collapse, two mechanisms occur simultaneously: curing the surrounding medium of the bubbles with extraordinary fast rate and the birth of daughter bubbles due to the IC bubble collapses. If the viscosity of the fluid were lower, the creation of the daughter bubbles would get easier. Therefore, in maxing ratio 10:1 in comparison with 13:1, more daughter bubbles are created and since the surrounding medium is being cured fast, some daughter bubbles can not be dissolved to the medium and stay in the cured medium and creates the pores in the structures. In higher ratios (such as 14:1, 15:1 and so on), the creation of daughter bubbles are prevented and the bubbles after collapse are disappeared in the medium due to the inertial flow of the medium toward inside of the collapsing bubbles.

3. Linear acoustics in high speed imaging tests

In the high speed imaging experiments, the ultrasound reflection from the platform is taking into account using linear acoustics. Assuming infinitesimal variation of density during the isentropic propagation of the focused ultrasound waves, the linear acoustic theory can be used. The Acoustics Module of COMSOL Multiphysics 5.4 software was used to investigate the linear behavior of the ultrasound wave propagation inside the build chamber and the locations of focal regions considering the reflection. The mass and momentum conservation equations

and energy equation are the employed governing equations for driving the linear wave equation in viscous medium as

$$\nabla.\left(-\frac{1}{\rho_c}\nabla p\right)-\frac{\omega^2 p}{\rho_c {c_c}^2}=0, \qquad (1)$$

where ω is the angular frequency (rad/s), p is the spatial pressure (Pa), c_c is the complex-valued speed of sound (m/s) expressed as $c_c=\omega/k$ where $k=\omega/c-i\alpha$ is the complex wave number in which c and α are the speed of sound and plane wave attenuation function respectively. The plane wave attenuation function, α (Np/m), in power law form is used as $\alpha=\alpha_0(f/f_0)^n$ where f, α_0 and n are transducer driving frequency (MHz) and the attenuation of material at f_0= 1 MHz, and a constant $n\in[0,2]$. ρ_c represents the complex-valued density defined as $\rho_c=\rho(c/c_c)^2$ where the ρ (kg/m^3) is the fluid density.

4. Nonlinear acoustics for bubble dynamics

Time dependent acoustic pressure at UAMR locations are predicted using non-linear acoustics for the later use in bubble dynamics. HITU_Simulator v2.0[5] is used to predict the propagated wave pressure inside the build chamber at UAMR. The software implements the wide-angle parabolic approximation of the Westervelt equation (wide-angle Khokhlov-Zabolotkaya-Kuznetsov, WAKZK) to calculate the pressure. This approximation results in one-way equation that takes into account spatial distribution of pressure of each harmonic, beam diffraction, interference effects and power-law frequency-dependence as[6]

$$\frac{\beta}{\rho c^4}\frac{\partial^2 p^2}{\partial t^2}+\nabla^2 p-\frac{1}{c^2}\frac{\partial^2 p}{\partial t^2}=\frac{2}{c}\frac{\partial}{\partial t}\big(\alpha(\omega)*p(\omega)\big), \qquad (2)$$

where p is the time-dependent pressure field in (Pa) and $p(\omega)$ is its frequency-domain representation, c sound speed (m/s), t time (s), β dimensionless nonlinear parameter, ρ density (kg/m^3), ω angular frequency (rad/s), ∇^2 is the Laplacian in cylindrical coordinate (cm^{-2}), $\alpha(\omega)$ is the attenuation function (cm^{-1}) which follows the power law form presented earlier, and $\alpha(\omega)*p(\omega)$ is the convolution of α and p. WAKZK neglects reflection and scattering which are crucial for predicting the locations of high pressure regions which create UAMRs. Therefore in our high speed imaging tests, linear acoustic is used instead.

Material related parameters for the multilayer medium domain for linear wave and WAKZK are as follows for water c=1486.6 m/s, ρ= 998 kg/m^3, α_0= 0.217 dB/m, n =2, β= 3.5; for PDMS c=1020 m/s, ρ= 965 kg/m^3, α_0= 147.66 dB/m, n =2, β= 4.5 and for the polystyrene platform c=2400 m/s, ρ= 1060 kg/m^3, α_0= 7.49 dB/m, n =2, β= 1. The thickness of the polystyrene barrier and platform of the build chamber is 1mm.

5. Bubble dynamics at UAMR

Since the printing material is the mixture of the monomer and the curing agent, the modified Keller-Miksis model for mixtures[7] is implemented in MATLAB by Eulerian finite difference

method to investigate the bubble dynamics at UAMR during the printing process. The Keller-Miksis radial oscillation of a bubble can be written as

$$\left(1-\frac{\dot{R}}{c_e}\right)R\ddot{R}+\frac{3}{2}\left(1-\frac{\dot{R}}{3c_e}\right)\dot{R}^2=\frac{1}{\rho_e}\left(1+\frac{\dot{R}}{c_e}\right)P_m+\frac{R}{\rho_e c_e}P_m\ , \quad (3)$$

where

$$P_m=\left(P_s+\frac{2\sigma_e}{R_0}\right)\left(\frac{R_0}{R}\right)^{3\gamma}+\left(1-\left(\frac{R_0}{R}\right)^{3\gamma}\right)P_v-\frac{\left(2\sigma_e+4\mu_e\dot{R}\right)}{R}-P(t)\ , \quad (4)$$

and R is the radius of the bubble, R_0 is the initial bubble radius, γ is the adiabatic index, P_s is the static pressure and P_v is the vapor pressure (can be neglected[8]). c_e, ρ_e, μ_e and σ_e are the equivalent sound speed, density dynamic viscosity, and surface tension of the mixture, respectively. $P(t)$ in Eq. 4 is the external pressure imposed by focused ultrasound and calculated via WAKZK (Eq. 3). In Eq. 3, the effects of bubble-bubble/particle interaction and gravity are neglected and it is assumed that the bubbles size are much larger than the size of particles suspended in the liquid. The input parameters for PDMS (10:1 mixing ratio) are c_e =1020 m/s, ρ_e = 965 kg/m^3, μ_e= 3.5 Pa.s. Bubble dynamics of different printing conditions are shown in Supplementary Fig. 13d and h and Supplementary Fig. 14i. Due to high viscosity of PDMS, R/R_0 is small (<1.045) and the concentration of the bubble size distribution is around R_0. The analyses of the bubble size distribution confirm this finding as the porosity size distribution are found out to be localized between 1μm to 5 μm.

6. Effects of environmental conditions, pressure and temperature, on DSP

Environmental conditions such as hydrostatic pressure or temperature could affect the printing resolution as well because the cavitation can be affected by these conditions. We investigated the effect on temperature by cooling the printing material (Supplementary Fig. 11) and we investigated the size of the printed spot on the platform. In the ambient temperature, 80 W power and 2.15 MHz leads to 1.3 mm spot diameter. However, by decreasing the temperature to -5 °C at the printing spot using thermoelectric coolers, the size of the spot reduced to 0.9 mm in diameter. In another setup, we investigated the effect of the static pressure on the spot size by applying external pressure to the printing medium (Supplementary Fig. 12). The spot size is reduced by increasing the static pressure using the hydraulic syringe. The gauge static pressure of 0, 0.9 kPa and 1.8 kPa resulted in 1.3 mm, 0.8 mm and 0.5 mm spot sizes, respectively. Although, as seen in these experiments, increasing the internal pressure and decreasing the temperature of the printing material could reach to a finer resolution, manipulating ultrasound frequency and power seem to be an easier way of tuning the resolution practically.

7. Synthesis and patterning application of DSP

In another application, DSP can be used for selective integration of functionalities such as electrical, optical, etc. to desired objects. Here we demonstrate simultaneous synthesis and

patterning of nano particles due to localized chemical activity of UAMR leading localized and selective patterning and making gold nano particle-PDMS composite. In this application, focused ultrasound waves induce almost instantaneous reduction of Gold (III) chloride trihydrate (HAuCl4.3H2O) with reducing agent present in PDMS to produce nano particles embedded in PDMS compared to the conventional heat assisted chemical systhesis process that takes hours. In addition, the instantaneous and localized ultrasound induced chemical synthesis of gold nano particles is used for fabricating a Localized Surface Plasmonic Resonance (LSPR) integrated mircrofluidic bio-sensing chip where DSP synthesizes and patterns gold nano islands (AuNIs) on a PDMS substrate.

Plasmonic property of AuNIs is used to detect extracellular vesicles or exosomes[9]. Exosomes or extracellular vesicles are nano sized particles containing mRNAs, microRNAs and lipids from their origin cells and have a critical role in cell to cell communication. A common method for exosome isolation and quantification is ultracentrifugation which is not practical for clinical settings[10]. In an alternative method[10], a specially synthesized polypetide (Vn96) is used to capture and quantify exosomes as shows in Supplementary Fig. 19a. A protocol (see Methods) to attached Vn96s to a PDMS substrate is illustrated in Supplementary Fig. 18. AuNIs are conventionally created on a glass or PDMS substrate by thermal convection and annealing process[9]. Here, in case of DSP, the desired pattern of AuNIs compatible with the designed micro chip is printed on the PDMS substrate directly as shown in Supplementary Fig. 19b where gold ions are reduced by polymer's cross-linking agent at UAMR instantly (Supplementary Movie 11). Supplementary Figs. 19c-e show a spiral, a maple leaf and a filled gear pattern of AuNIs which demonstrate the flexibility of DPS in printing customized patterns for bio-sensing purposes (Supplementary Movies 12 and 13). The designed microchip with microfluidic channels (500 µm by 150 µm) and chambers (Dia. 5 mm) is illustrated in Supplementary Fig. 19f where the localized surface plasmon resonance (LSPR) band of AuNIs is measured by a spectrophotometer. Supplementary Fig. 19g shows the fabricated micro chip while exosomes are passed through the chip. A plasmonic wavelength shift (6 nm) towards longer wavelength, due to the interaction between the immobilized Vn96 on gold nano islands and exosomes from breast cancer cells MCF7, is detected as shown in Supplementary Fig. 19h. The patterned gold nano islands on the PDMS substrate create a surface and sub-surface gold nanoparticle-polymer composite locally. The diffusion depth, d_p, of the nanoparticles are about a few micrometer by conventional heat and annealing methods[11]. However, X-ray photoelectron spectroscopy (XPS) measurement of the printed patterns shows that using DSP, gold nano particles could diffuse a few hundreds of micrometers from the surface of the polymer as shown Supplementary Fig. 19i.

Supplementary Figures

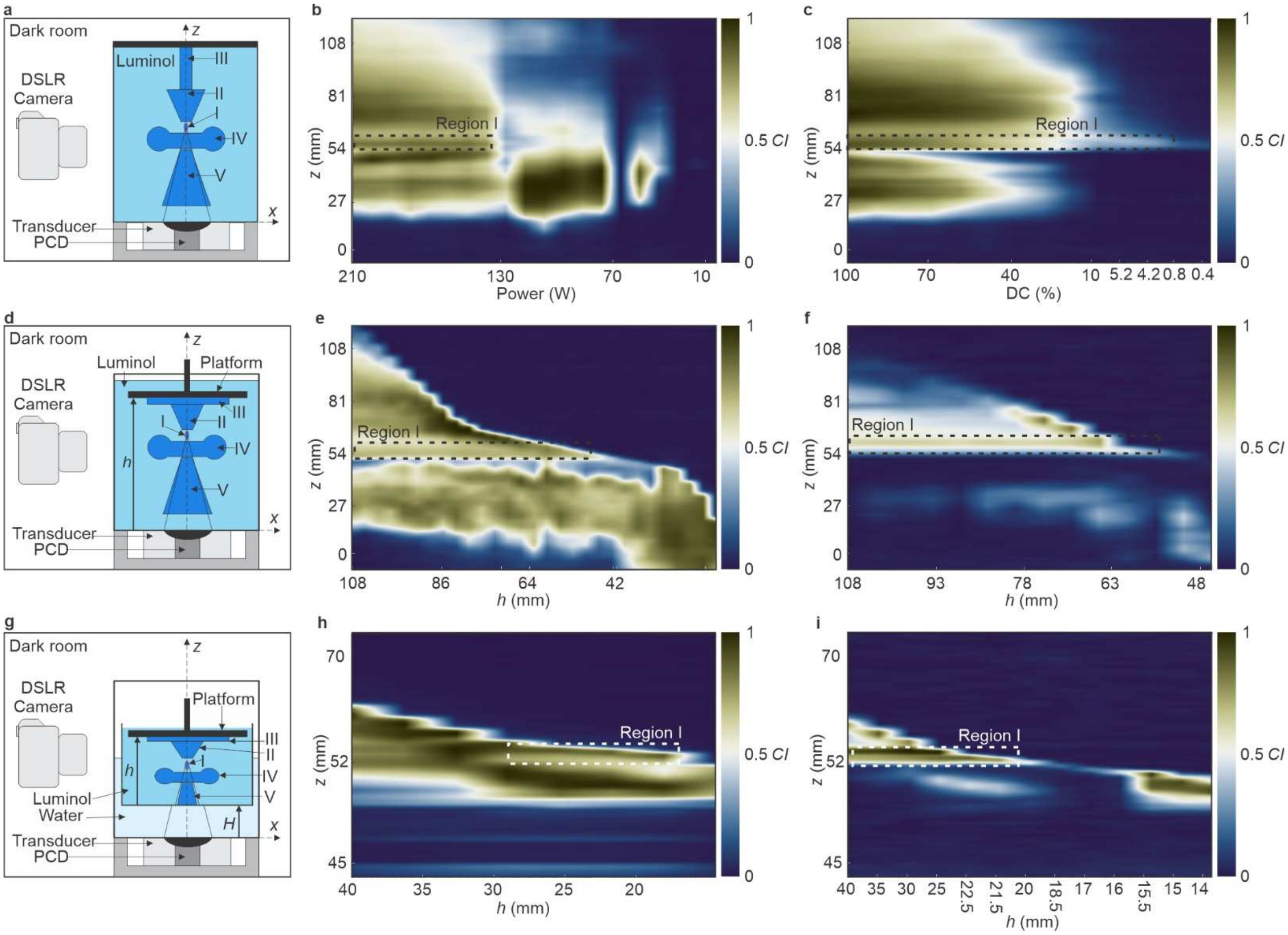

Supplementary Fig. 1| The SCL (Sonochemiluminescnce) experiments leading to DSP concept. **a**, SCL setup for direct exposure to luminol. **b** and **c**, ASDS (Axial SCL Distribution Spectrum) diagrams for varying power & constant DC (Duty Cycle) 100% and varying DC & constant power 210 W, respectively, the setup shown in **a**. **d**, SCL setup for direct exposure to luminol with platform presence and varying *h*. **e** and **f**, ASDS diagram for varying *h* and constant power 210 W with DC 100% and DC 30%, respectively, for the setup shown in **d**. **g**, SCL setup for indirect exposure to luminol with platform presence, varying *h* and constant *H*=30 mm. **h** and **i,** ASDS diagram for varying *h* and constant power 210 W with DC 100% and DC 30%, respectively, for the setup shown in **g.** For the SCL tests, transducer H-148 with driving frequency of f = 2.15 MHz is used. *CI* is the normalized light intensity along z axis calculated from SCL images in Supplementary Figs 2-7. (PCD: Passive Cavitation Detector, DSLR: Digital Single-Lens Reflex, CI: Color Intensity)

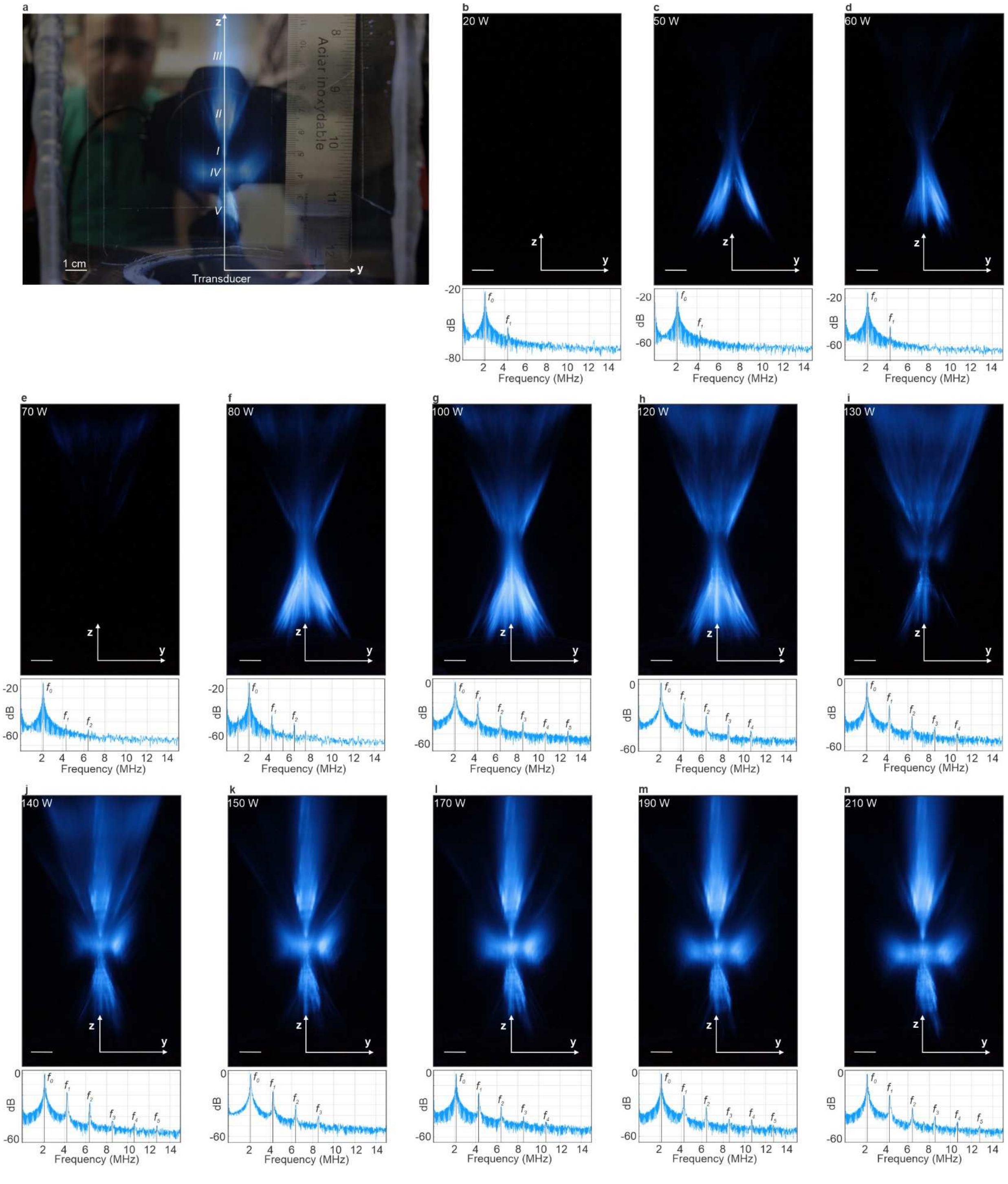

Supplementary Fig. 2| SCL experiments for setup shown in Fig. 2a. a, The focused transducer transmitting ultrasound directly to the luminol solution and the blue light patterns of SCL captured via the DSLR camera. **b-n,** SCL patterns captured via the DSLR camera and their corresponding FFT of PCD signal of focal (region I) under different electrical powers. Harmonic frequencies are specified on the FFT diagrams. Experiment conditions: varying power, DC = 100%, f = 2.15 MHz, ISO 3200 , exposure time 30 s.

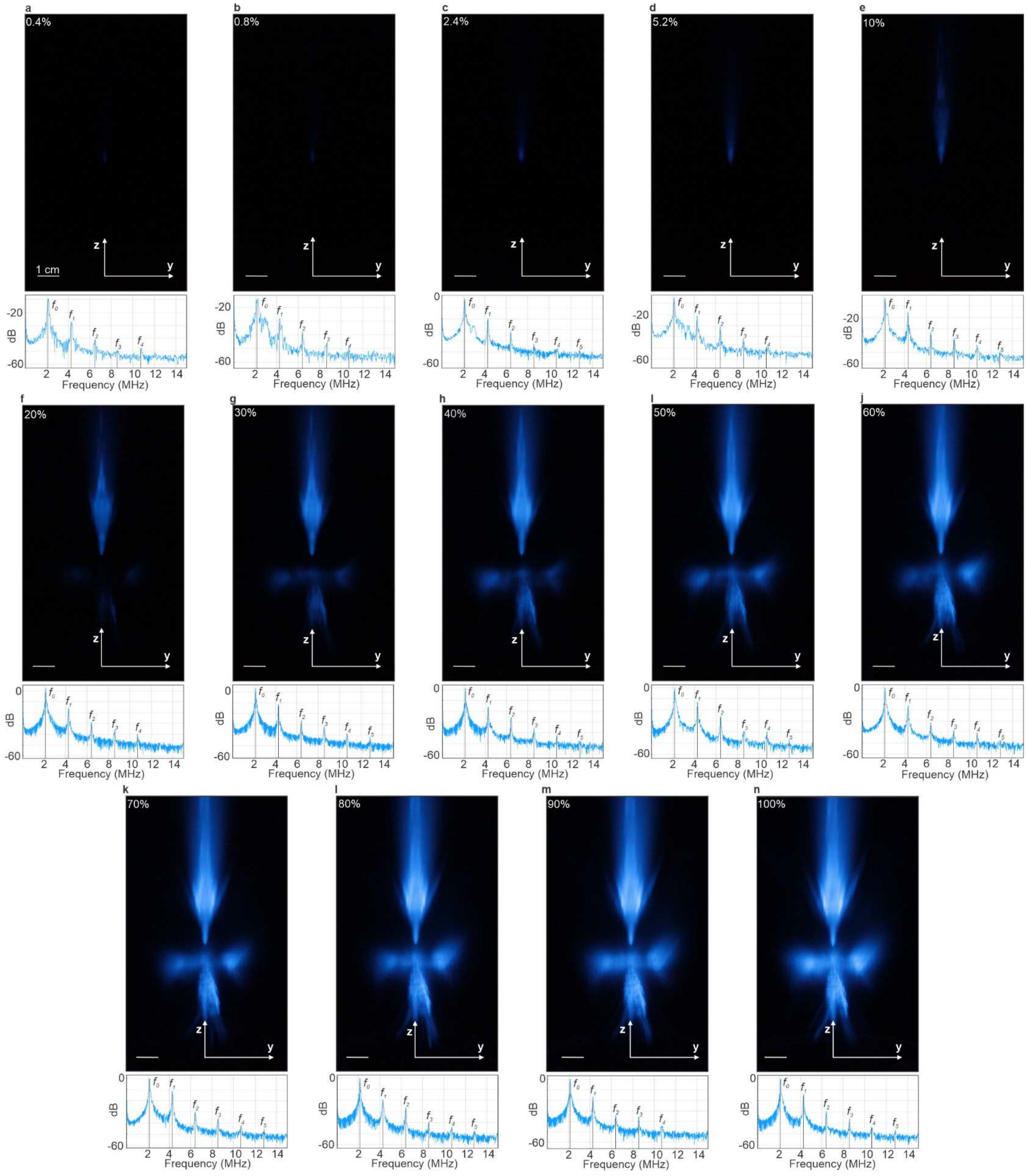

Supplementary Fig. 3| SCL experiments for setup shown in Fig. 2a. a-n, SCL patterns captured using the DSLR camera and their corresponding FFT of PCD signal of focal (region I) under different DCs. Harmonic frequencies are specified on the FFT diagrams. Experiment conditions: power = 210 W, varying DC, f = 2.15 MHz, ISO 3200 and exposure time 30 s.

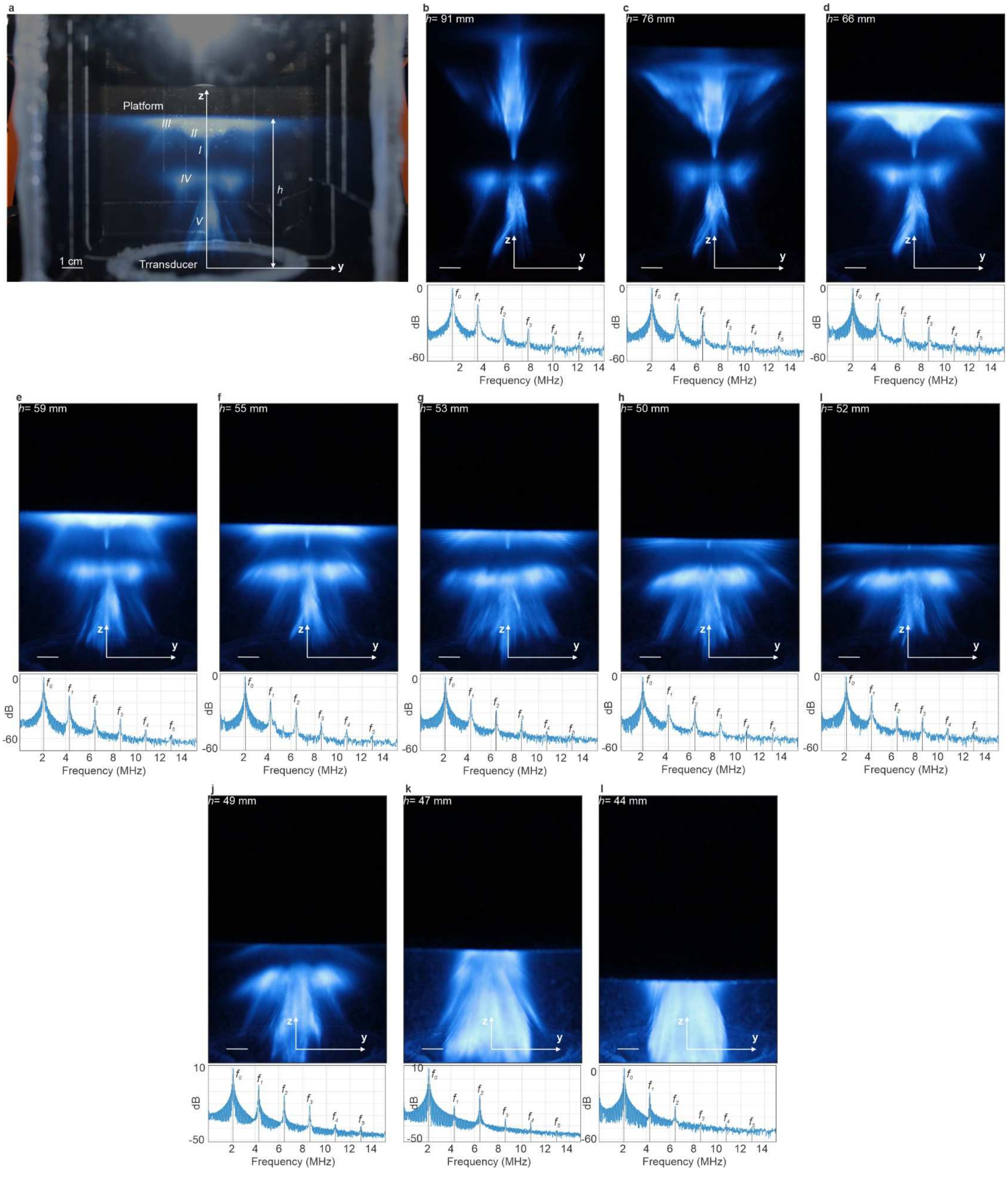

Supplementary Fig. 4| SCL experiments for setup shown in Fig. 2d. a, The HIFU transducer transmitting ultrasound directly to the luminol solution with presence of a platform with different height, *h*, and the blue light patterns of SCL captured using the DSLR camera. **b-i,** SCL patterns captured via the DSLR camera and their corresponding FFT of PCD signal for different *h*. Harmonic frequencies are specified on the FFT diagrams. Experiment conditions: power = 210 W, DC = 100%, f = 2.15 MHz, ISO 3200 and exposure time 30 s.

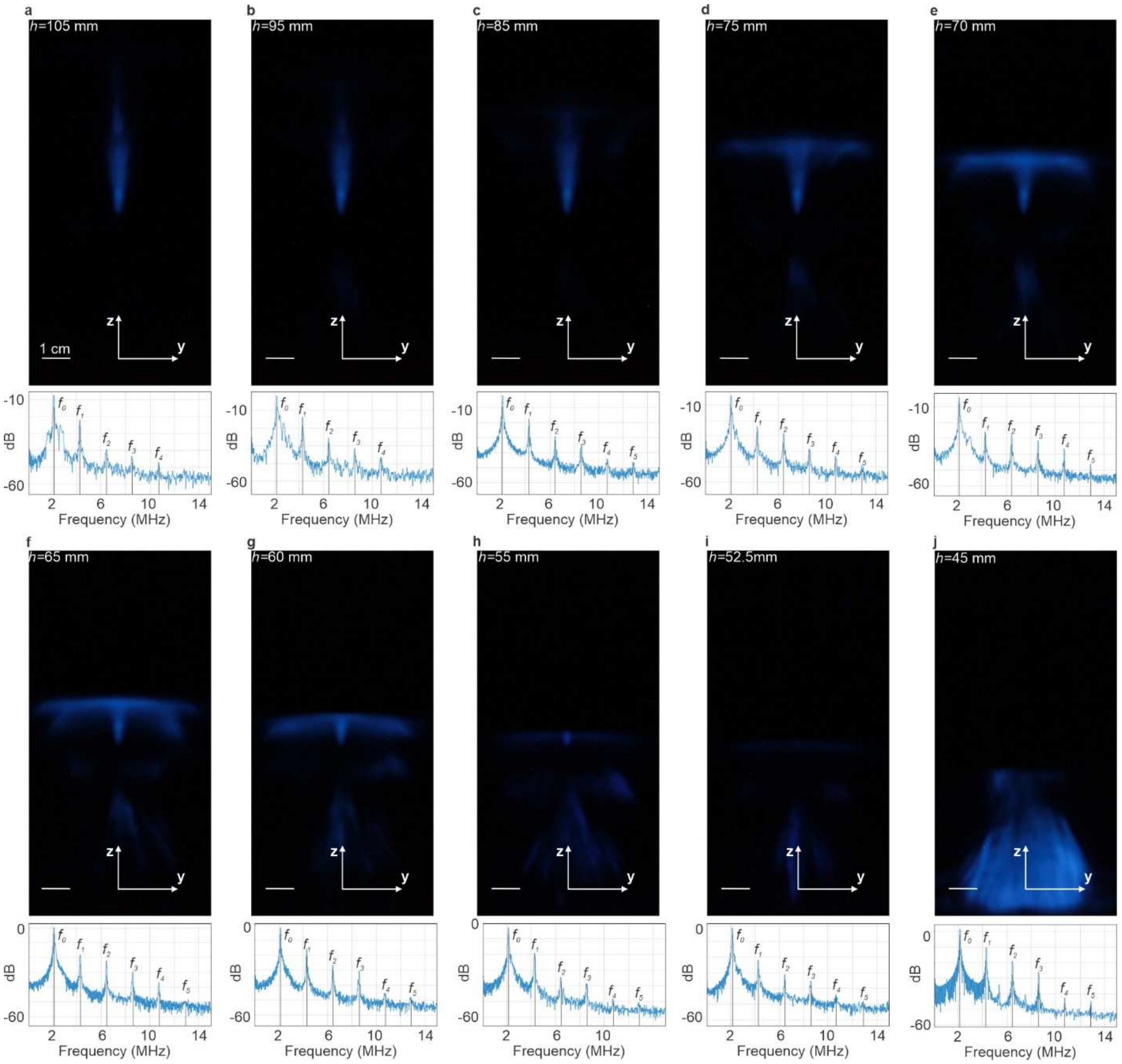

Supplementary Fig. 5| SCL experiments for setup shown in Fig. 2d. a, The HIFU transducer transmitting ultrasound directly to the luminol solution with presence of a platform with different height, *h*, and the blue light patterns of SCL captured using the DSLR camera. **b-i,** SCL patterns captured via the DSLR camera and their corresponding FFT of PCD signal for different *h*. Harmonic frequencies are specified on the FFT diagrams. Experiment conditions: power = 210 W, DC = 30%, *f* = 2.15 MHz, ISO 3200 and exposure time 30 s.

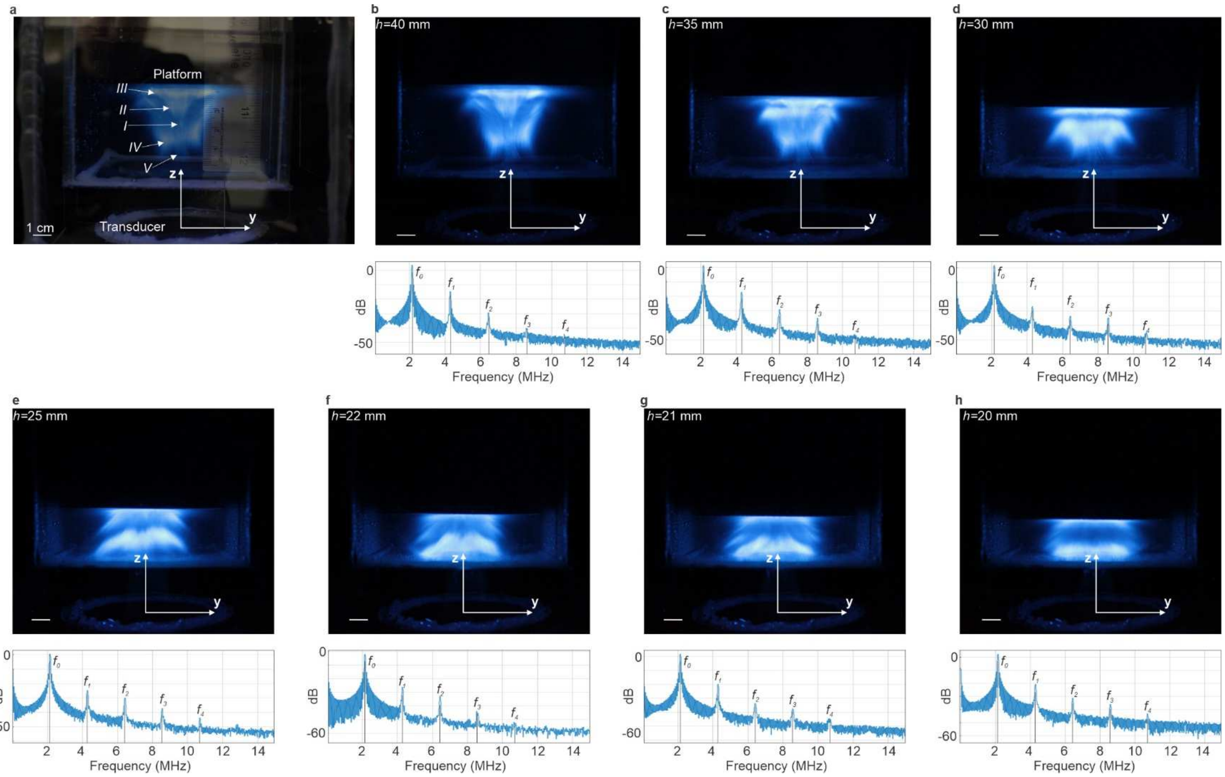

Supplementary Fig. 6| SCL experiments for setup shown in Fig. 2g. a, The HIFU transducer transmitting ultrasound to the luminol chamber passing though the polystyrene shell of the chamber with presence of a platform with different height, *h*, and the blue light patterns of SCL captured using the DSLR camera. **b-h,** SCL patterns captured via the DSLR camera and their corresponding FFT of PCD signal for different *h*. Harmonic frequencies are specified on the FFT diagrams. Experiment conditions: power = 210 W, DC = 100%, *f* = 2.15 MHz, ISO 3200 and exposure time 10 s.

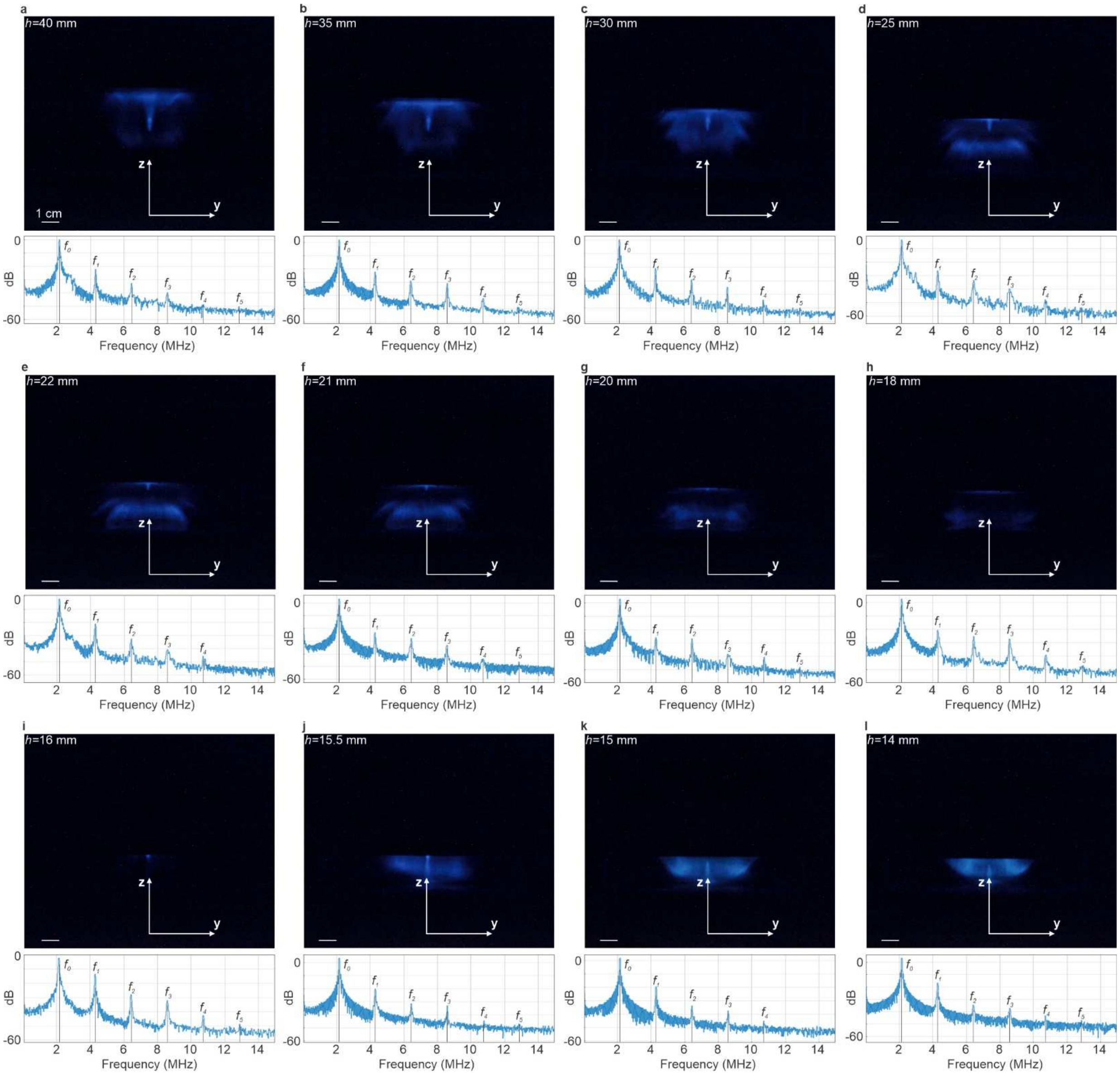

Supplementary Fig. 7| SCL experiments for setup shown in Fig. 2g. a, The HIFU transducer transmitting ultrasound to the luminol chamber passing though the polystyrene shell of the chamber with presence of a platform with different height, *h*, and the blue light patterns of SCL captured using the DSLR camera. **b-h,** SCL patterns captured via the DSLR camera and their corresponding FFT of PCD signal for different *h*. Harmonic frequencies are specified on the FFT diagrams. Experiment conditions: power = 210 W, DC = 30%, f = 2.15 MHz, ISO 3200 and exposure time 10 s.

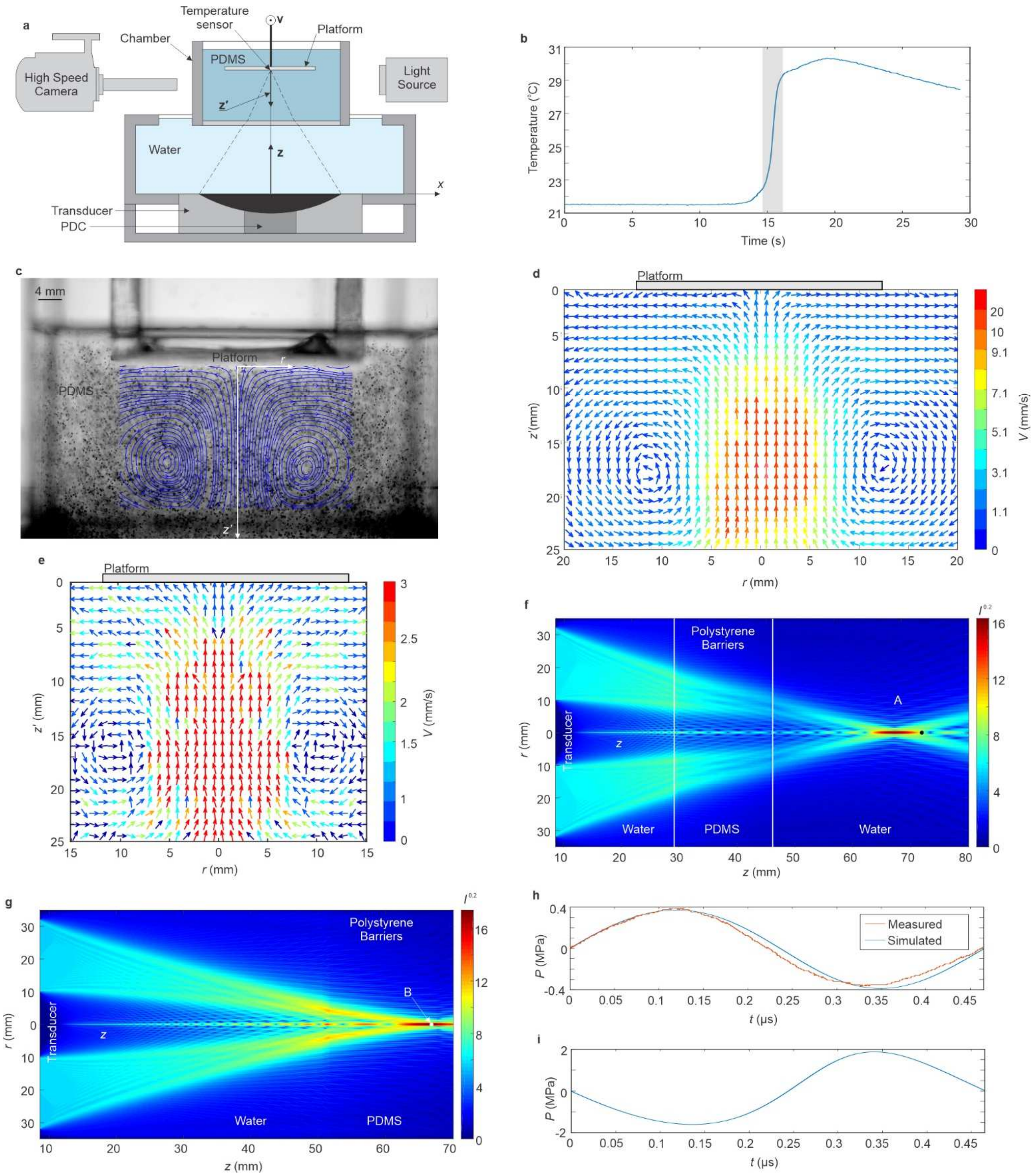

Supplementary Fig. 8| Working conditions (pressure, temperature and streaming) in the printing chamber. a, Schematic view of the setup for temperature and particle tracing experiments. **b,** Printing material's temperature at the location of the temperature sensor while a straight line is printed (the sensor is placed at the center of the line). The highlighted region represents the period when the material is solidified on the sensor (printing condition: power= 210 W (extremely high), v = 300mm/min, f = 2.15 MHz, DC = 100% and transducer type: H-148). **c**, Stream lines. **d**, streaming velocity in the build chamber for the extremely high electrical power of 210 W. **e,** streaming velocity for a real printing case with electrical power of 20 W and 100 % DC. **f** and **g**, Simulated intensity , I (W/m^2), to the power of 0.2 for the cases where the focal region is located at the back of the build chamber (the polystyrene platform and barrier) and at the platform, respectively. **h**, Measured and simulated temporal pressure at point A shown in **f**. **h**, Simulated temporal pressure at point B shown in **g**. (ultrasound conditions in **f**-**i**, power = 20 W, f = 2.15 MHz, DC = 100 % and transducer type: H-148) (PDMS: Polydimethylsiloxane)

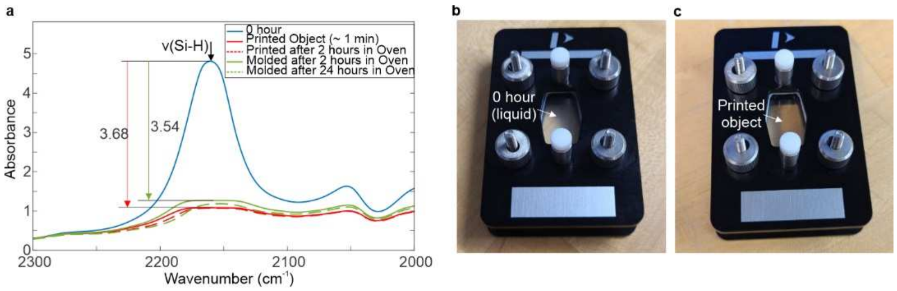

Supplementary Fig. 9| IR spectrum measurement for curing rate identification in DSP printed vs. molded parts. a, IR Spectrum of mixing ratio of 20:1. **b and c,** The resin mixture at 0 hour and printed part, respectively, in a liquid transmission cells for IR measurements.

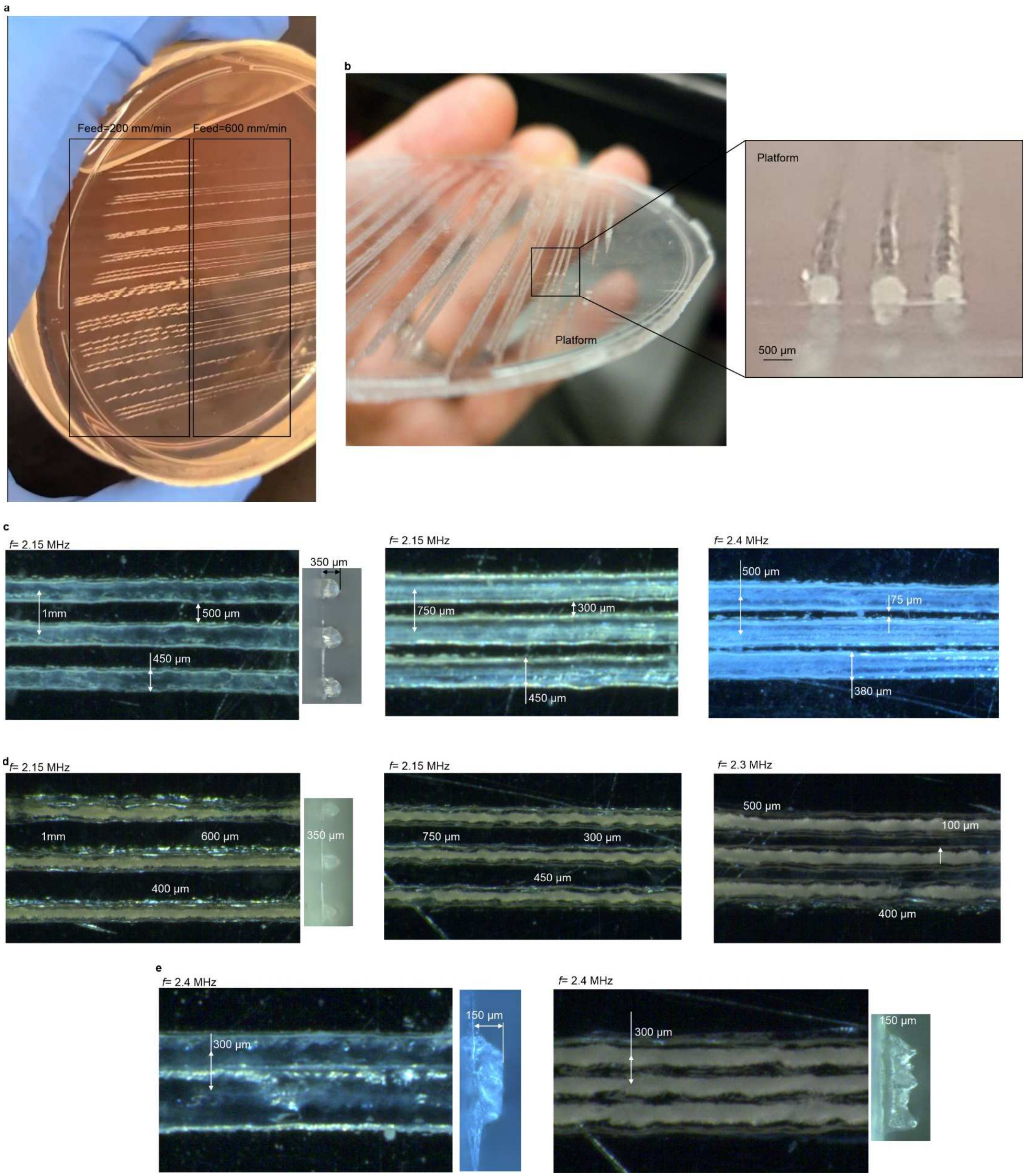

Supplementary Fig. 10| Characterizing XY printing resolution of DSP by printing straight lines. a and b, Printed lines with Sylgard-184 mixing ratios of 10:1 (porous) and 15:1 (transparent), respectively., **c and d**, Microscopic images of printed distinguishable lines and gaps with the ratio of 15:1 and 10:1, respectively. **e,** Undistinguishable lines with 300 µm separation (center to center) for mixing ratios 15:1 (left) and 10:1 (right).

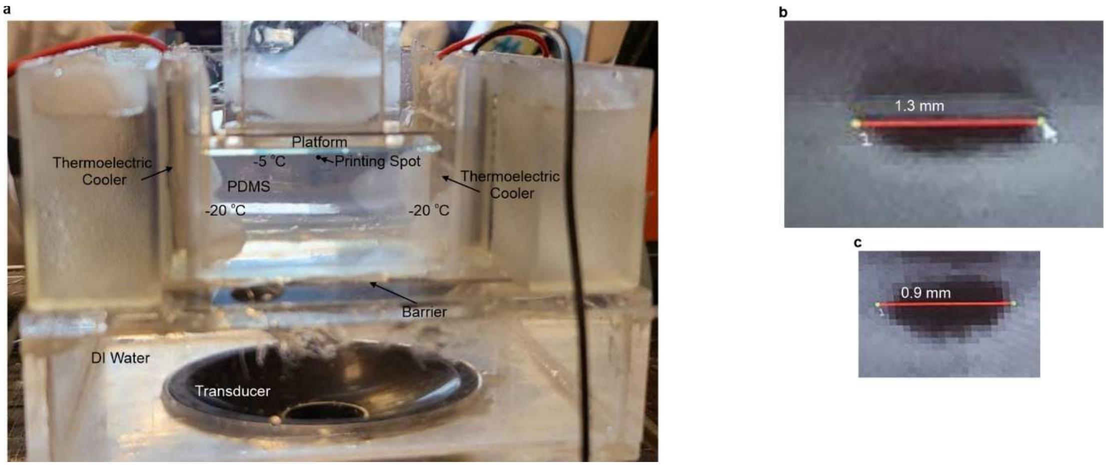

Supplementary Fig. 11| Investigation of temperature decrease on the printed spot size. a, Experimental setup. **b,** printed spot in ambient temperature. **c,** printed spot at -5 ºC. (PDMS: Polydimethylsiloxane)

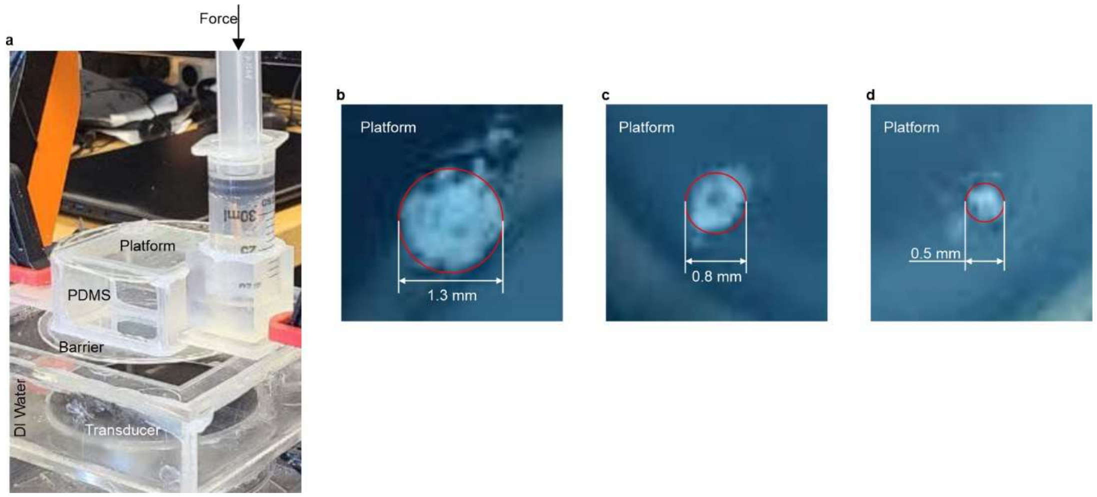

Supplementary Fig. 12| Investigation of static pressure increase on the printed spot size. a, Experimental setup. **b-d,** printed spot sizes for static pressures of 0, 0.9 kPa and 1.8 kPa , respectively. (PDMS: Polydimethylsiloxane)

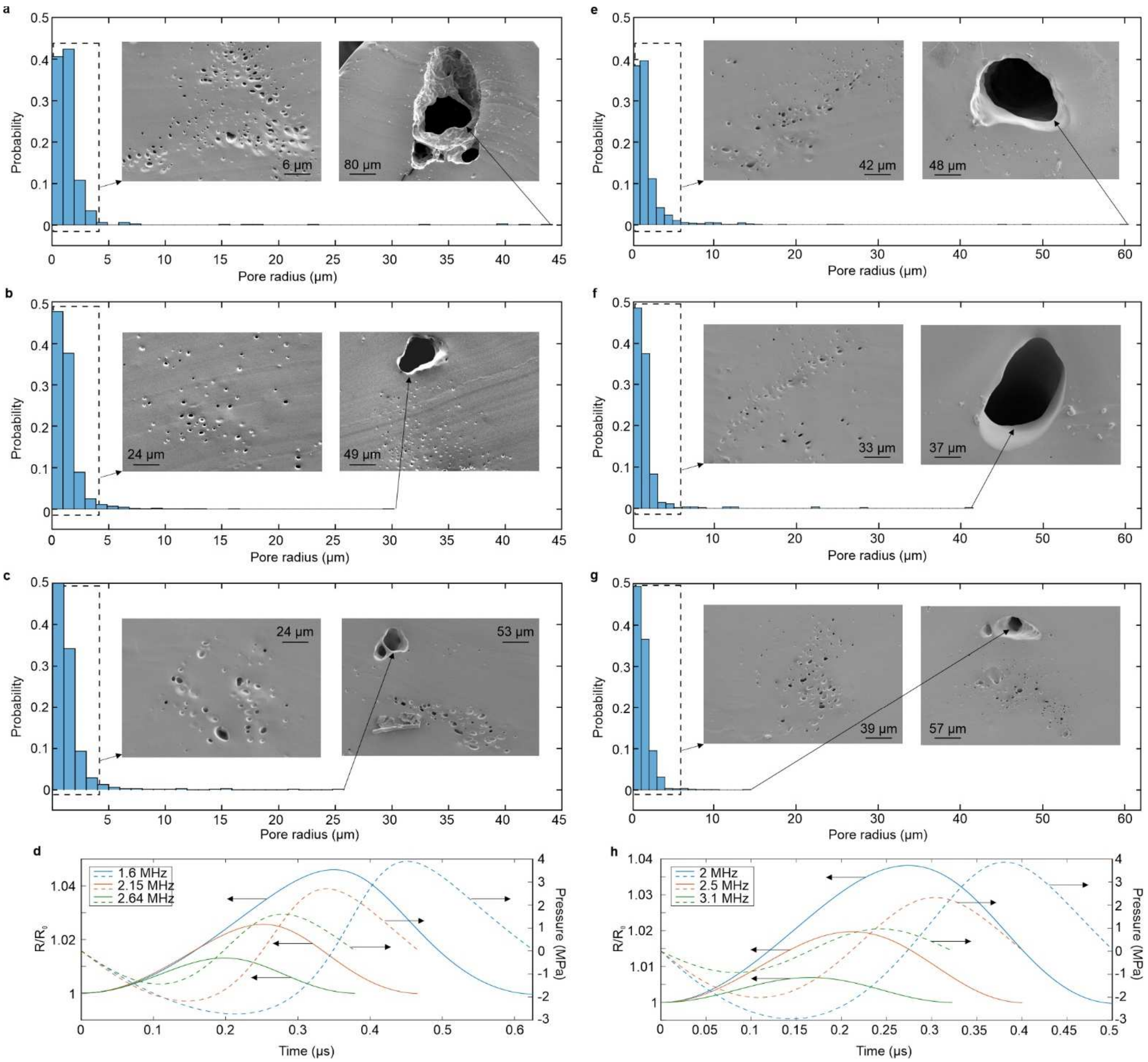

Supplementary Fig. 13| The histogram diagram of the pore size analysis of SEM pictures from the printed walls with different ultrasound frequencies for two transducers H-148 and H-316 (sample SEM pictures are shown for the indicated location on the histogram diagram). a-c, Pore size distribution for frequencies 1.6, 2.15 (natural frequency of H-148) and 2.64 MHz, respectively. **d**, Simulated non-linear pressure and R/R_0 ratio on the printing platform for a-c. **e-g,** Pore size distribution frequencies 2, 2.5 (natural frequency of H-316) and 3.1 MHz, respectively. **h**, Simulated non-linear pressure and R/R_0 ratio on the printing platform for e-g. Printing conditions: power = 40 W, DC = 100% and v = 350 mm/min.

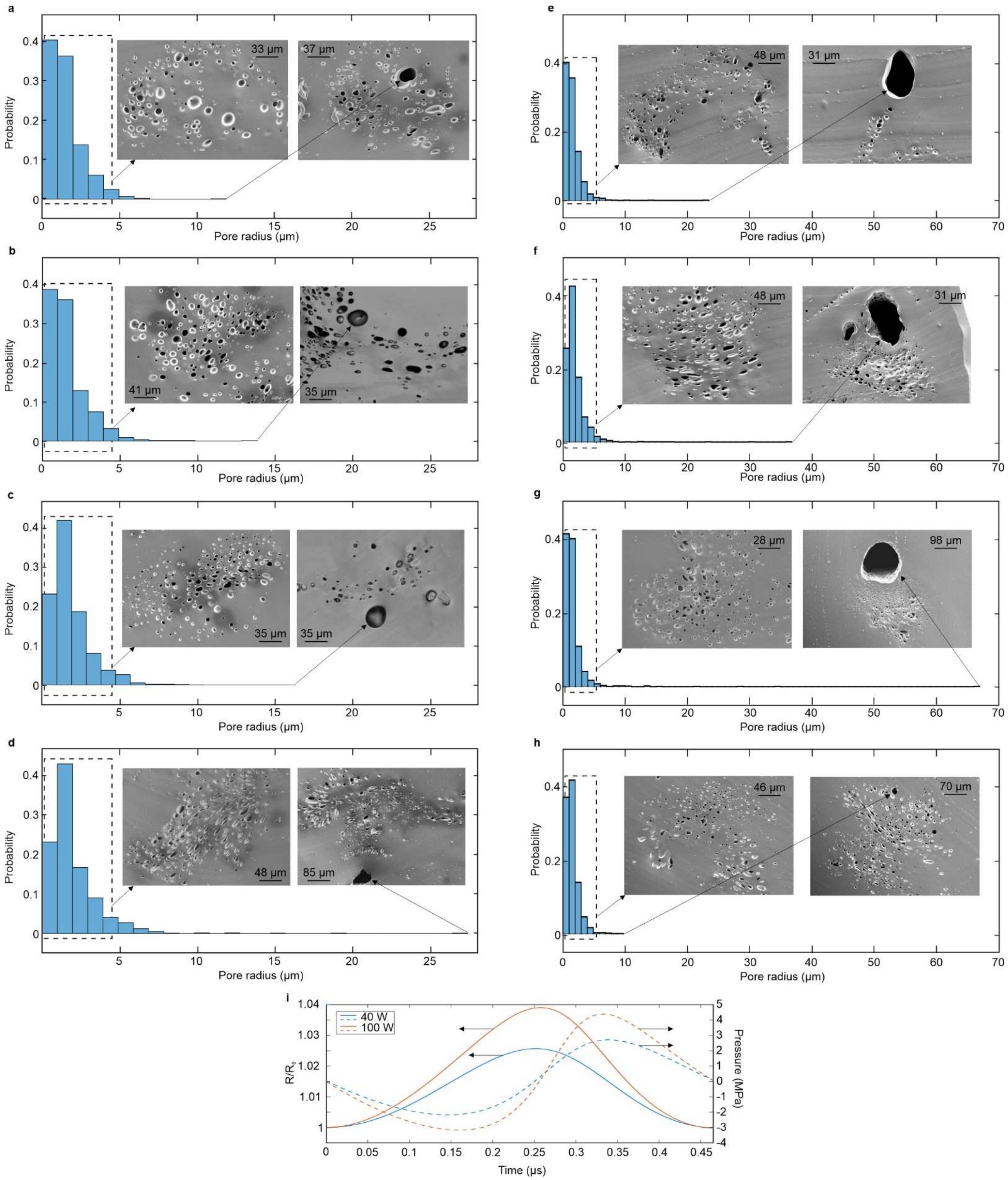

Supplementary Fig. 14| The histogram diagram of the pore size analysis of SEM pictures from the printed walls with varying DCs for the transducer H-148 (sample SEM pictures are shown for the indicated location on the histogram diagram). a-d, Pore size distribution at power 40 W for DCs 30%, 50%, 70% and 100%, respectively. **e-h,** Pore size distribution at power 100 W for DCs 30%, 50%, 70% and 100%, respectively. **h**, Simulated non-linear pressure and R/R_0 ratio on the printing platform for powers 40 W and 100W. Printing conditions: f = 2.15 MHz and v = 350 mm/min.

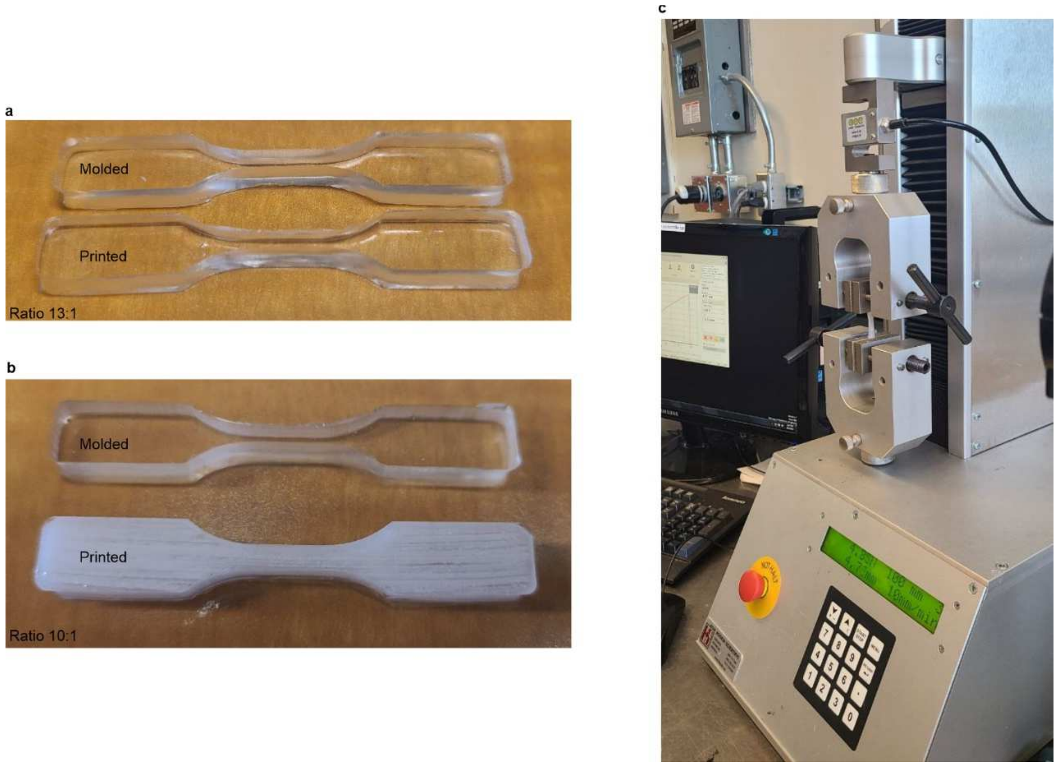

Supplementary Fig. 15| Mechanical characteristic of DSP printed vs. molded parts . a and b, Printed and molded dog-bone specimens for the tensile test for mixing ratios of 13:1 and 10:1, respectively. **h**, Z5, Hoskin Scientific test stand.

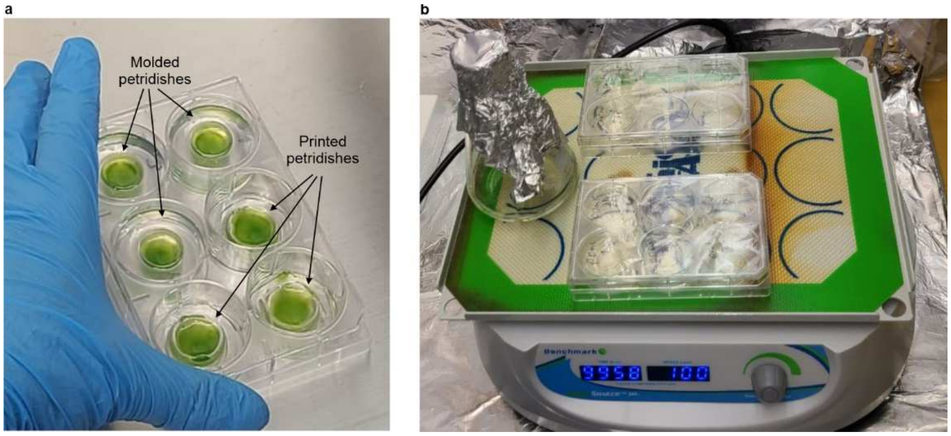

Supplementary Fig. 16| Chlamydomonas reinhardtii cell culture on printed and molded petridishes. a, Molded and printed petridishes at day one of a 7-day cell culture. **b,** The culture dish placed on the orbital shaker at day one.

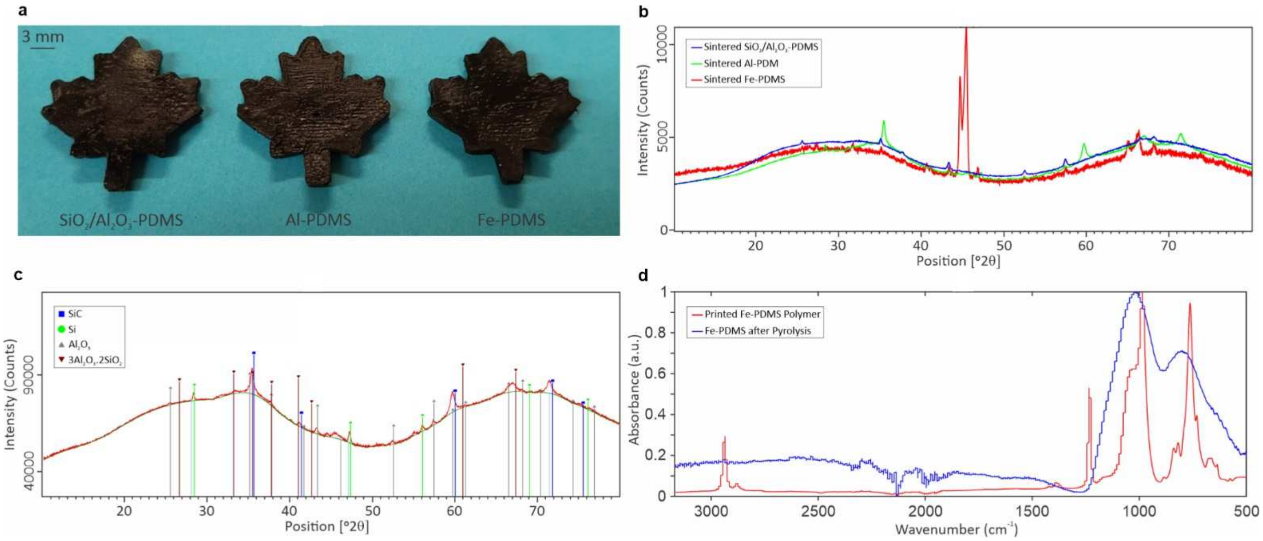

Supplementary Fig. 17| Sintered opaque printed material and characterization. a, Sintered ceramic maple leaves from printed opaque micro/nano composite materials of PDMS and Silica/Alumina, Aluminum and Iron. **b**, XRD patterns of the ceramic parts in **a. c**, Crystalline peak identification in Al-PDMS ceramic. **d**, FTIR spectra of Fe-PDMS after printing (polymeric part) and after pyrolysis (ceramic part). (PDMS: Polydimethylsiloxane)

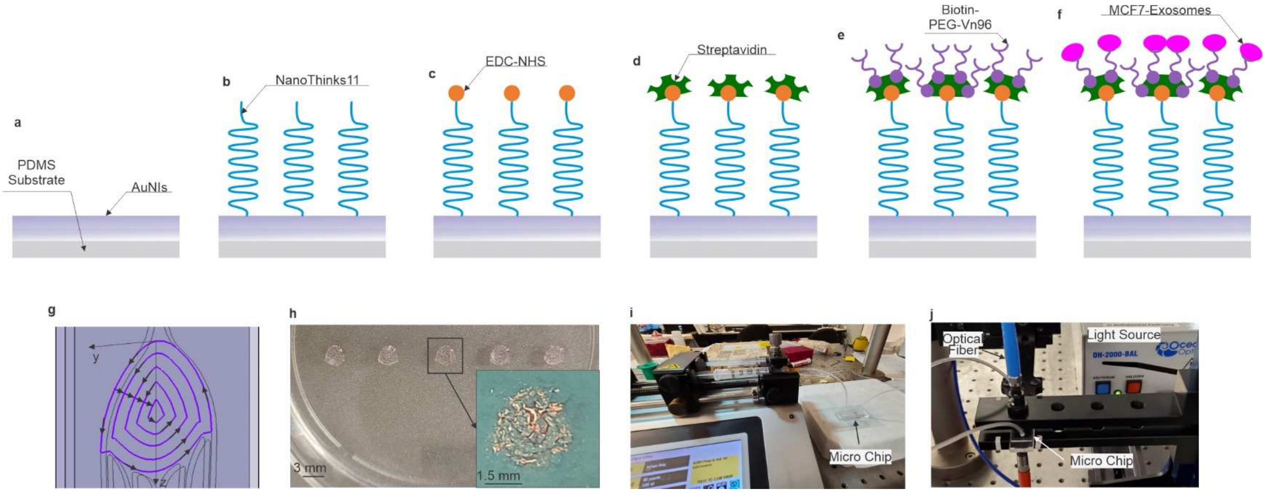

Supplementary Fig. 18| Synthesizing and pattering of Gold nano islands in biosensing micro chip. a-f, Biosensing protocol for detection of exosomes. **g**, Computer generated patterns/paths for UAMR to follow. **h**, Patterned AuNIs. **i**, The microchip connected to the syringe pump. **j**, LSPR of the collection chamber is measured by the spectrometer.(PDMS: Polydimethylsiloxane)

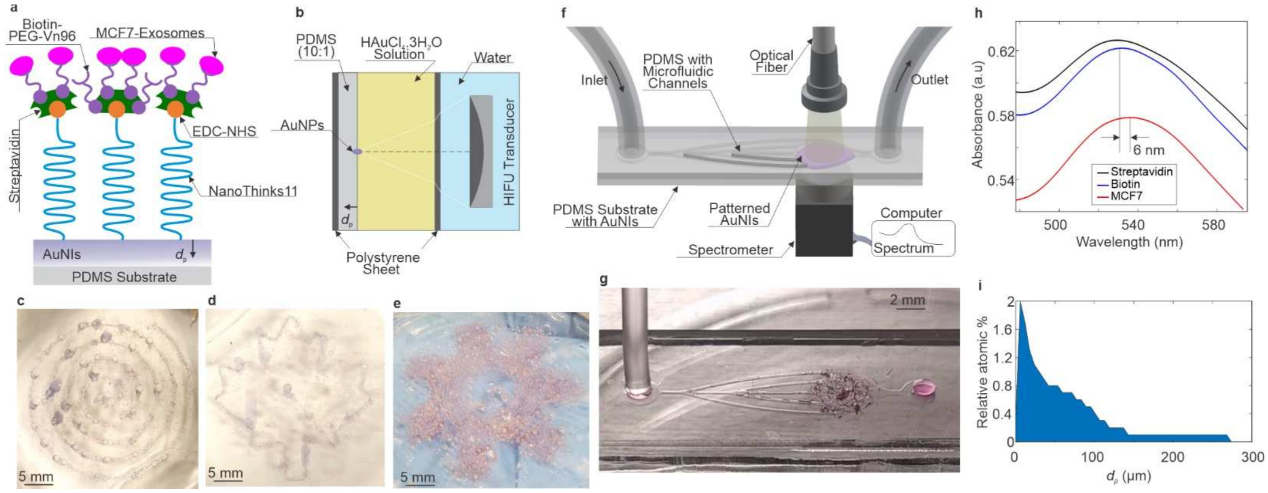

Supplementary Fig. 19| Synthesis and patterning application of DSP. a, A method to capture exosomes on AuNIs. **b**, Schematic view of synthesising and patterning gold nano particles using DSP. **c-e**, Synthesized and patterned AuNIs in the forms of spiral and maple leaf and gear patterns. **f**, Schematic of the fabricated micro chip and measurement setup for capturing and detection exosomes. **g**, Fabricated micro chip when exosomes are passing through the its microfluidic channels. **h**, Absorption spectra for the last stage of the protocol described in Method. **i**, XPS measurement of gold atomic concentration vs. d_p shown in **a** and **b**.

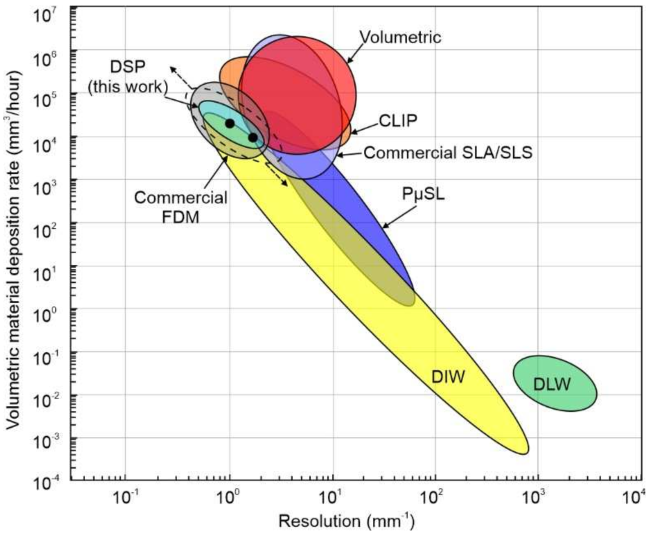

Supplementary Fig. 20| Holistic comparison of printing performance (disposition rate vs. resolution (1/(2×δ) where δ is feature size)) among existing heat and light based AM methods[12,13] and this work (DSP). The dashed boundary and connected arrows show the potential development region and its expanding direction of DSP. (DIW: Direct Ink Writing, SLA: Stereolithography, FDM: Fused Deposition Modeling, PμSL: Projection Micro-Stereolithography, CLIP: Liquid Interface Printing, DSP: Direct Sound Printing)

References

1. The Dow Chemical Company. SYLGARD™ 184 Silicone Elastomer APPLICATIONS. *Silicone Elastomer Tech. Data Sheet* 4 (2017).

2. Dow, T. H. E. *et al. SYLGARD™ 184 Silicone Elastomer Curing Agnet.* (2018).

3. Yasui, K. *SPRINGER BRIEFS IN MOLECULAR SCIENCE Acoustic Cavitation and Bubble Dynamics*. (2018).

4. Li, Y. *et al.* Evaluation of the properties of daughter bubbles generated by inertial cavitation of preformed microbubbles. *Ultrason. Sonochem.* **72**, 105400 (2021).

5. Soneson, J. E. A user-friendly software package for HIFU simulation. *AIP Conf. Proc.* **1113**, 165–169 (2009).

6. Soneson, J. E. Extending the Utility of the Parabolic Approximation in Medical Ultrasound Using Wide-Angle Diffraction Modeling. *IEEE Trans. Ultrason. Ferroelectr. Freq. Control* **64**, 679–687 (2017).

7. Huang, S. & Mohamad, A. A. Modeling of cavitation bubble dynamics in multicomponent mixtures. *J. Fluids Eng. Trans. ASME* **131**, 0313011–0313015 (2009).

8. Jiang, L., Ge, H., Liu, F. & Chen, D. Investigations on dynamics of interacting cavitation bubbles in strong acoustic fields. *Ultrason. Sonochem.* **34**, 90–97 (2017).

9. Badilescu, S., Raju, D., Bathini, S. & Packirisamy, M. Gold nano-island platforms for localized surface plasmon resonance sensing: A short review. *Molecules* **25**, (2020).

10. Bathini, S., Raju, D., Badilescu, S. & Packirisamy, M. Microfluidic Plasmonic Bio-Sensing of Exosomes by Using a Gold Nano-Island Platform. *Int. J. Biomed. Biol. Eng.* **12**, 236–239 (2018).

11. Bathini, S., Raju, D., Badilescu, S. & Packirisamy, M. EFFECT OF CROSS-LINKING AND THERMAL BUDGET ON PLASMONIC SENSING AND SUB-SURFACE SEGREGATION OF IN-SITU SYNTHESIZED GOLD IN POLYMER-NANO COMPOSITE. *J. Nanoparticle Res.* (2020).

12. Shusteff, M. *et al.* One-step volumetric additive manufacturing of complex polymer structures. *Sci. Adv.* **3**, (2017).

13. Ultimaker. https://ultimaker.com/3d-printers
