# Supplementary material for: Direct sound printing
Source: Nat Commun. 2022 Apr 6;13:1800. doi: 10.1038/s41467-022-29395-1 (PMC8986813; doi:10.1038/s41467-022-29395-1)
Supplement: Supplementary file 3 — Description of Additional Supplementary Files [file 41467_2022_29395_MOESM3_ESM.pdf]

Description of Additional Supplementary Files

File Name: Supplementary Movie 1
Description: DSP in Action, printing porous and transparent structures.

File Name: Supplementary Movie 2
Description: High Speed Imaging Videos of Fig. 2e, Combination of 12 static printing tests for different h related to Fig. 2e where only the test at h=30mm is illustrated.

File Name: Supplementary Movie 3
Description: High Speed Imaging Video of Fig. 2f, 7x magnification high speed imaging footage of the static printing test at h=22mm. This video is the footage illustrated in Fig. 2f.

File Name: Supplementary Movie 4
Description: High Speed Imaging of DSP while printing porous structure in Fig. 3.

File Name: Supplementary Movie 5
Description:
High Speed Imaging of DSP while printing transparent structure in Fig. 3.

File Name: Supplementary Movie 6
Description: Particle Image Velocimetry (PIV) of extremely high power vs printing power in DPS as in Supplementary Fig. 8d and e.

File Name: Supplementary Movie 7
Description: DSP/RDP of Opaque Silica/Alumina-PDMS Colloidal Solution.

File Name: Supplementary Movie 8
Description: DSP/RDP of Opaque Fe-PDMS Colloidal Solution

File Name: Supplementary Movie 9
Description: DSP/RDP in action while real porcine tissue, containing muscle, fat and skin.

File Name: Supplementary Movie 10
Description: DSP/RDP in action while a tissue phantom.

File Name: Supplementary Movie 11
Description: AuINs are patterned and Synthesized using DSP for the surface of a collecting chamber of a biosensing platform.

File Name: Supplementary Movie 12
Description: Patterning and Synthesizing AuNIs using DSP in the Shape of a Maple Leaf to demonstrate the flexibility of DSP in patterning geometry.

File Name: Supplementary Movie 13
Description: Patterning and Synthesizing AuNIs using DSP in the Shape of a spiral to demonstrate the flexibility of DSP in patterning geometry.
