# Supplementary material for: Direct sound printing
Source: Nat Commun. 2022 Apr 6;13:1800. doi: 10.1038/s41467-022-29395-1 (PMC8986813; doi:10.1038/s41467-022-29395-1)
Supplement: Supplementary file 2 — Peer Review File [file 41467_2022_29395_MOESM2_ESM.pdf]

Editorial Note: This manuscript has been previously reviewed at another journal that is not operating a transparent peer review scheme. This document only contains reviewer comments and rebuttal letters for versions considered at Nature Communications.
